# Mutational processes of tobacco smoking and APOBEC activity generate protein-truncating mutations in cancer genomes

**DOI:** 10.1126/sciadv.adh3083

**Published:** 2023-11-03

**Authors:** Nina Adler, Alexander T. Bahcheli, Kevin C. L. Cheng, Khalid N. Al-Zahrani, Mykhaylo Slobodyanyuk, Diogo Pellegrina, Daniel Schramek, Jüri Reimand

**Affiliations:** ^1^ Computational Biology Program, Ontario Institute for Cancer Research, Toronto, ON, Canada.; ^2^ Department of Molecular Genetics, University of Toronto, Toronto, ON, Canada.; ^3^ Department of Medical Biophysics, University of Toronto, Toronto, ON, Canada.; ^4^ Lunenfeld-Tanenbaum Research Institute, Toronto, ON, Canada.

## Abstract

Mutational signatures represent a genomic footprint of endogenous and exogenous mutational processes through tumor evolution. However, their functional impact on the proteome remains incompletely understood. We analyzed the protein-coding impact of single-base substitution (SBS) signatures in 12,341 cancer genomes from 18 cancer types. Stop-gain mutations (SGMs) (i.e., nonsense mutations) were strongly enriched in SBS signatures of tobacco smoking, APOBEC cytidine deaminases, and reactive oxygen species. These mutational processes alter specific trinucleotide contexts and thereby substitute serines and glutamic acids with stop codons. SGMs frequently affect cancer hallmark pathways and tumor suppressors such as *TP53*, *FAT1*, and *APC*. Tobacco-driven SGMs in lung cancer correlate with smoking history and highlight a preventable determinant of these harmful mutations. APOBEC-driven SGMs are enriched in YTCA motifs and associate with *APOBEC3A* expression. Our study exposes SGM expansion as a genetic mechanism by which endogenous and carcinogenic mutational processes directly contribute to protein loss of function, oncogenesis, and tumor heterogeneity.

## Teaser

Mutational processes of tobacco smoking and APOBEC disrupt gene function in cancer by adding premature stop codons.

## INTRODUCTION

Cancer is driven by a few somatic mutations that enable oncogenic properties of cells; however, most mutations in cancer genomes are functionally neutral passengers (*1*, *2*). Somatic mutations are caused by endogenous and exogenous mutational processes with complex context- and sequence-specific activities that collectively mark tumor evolution and exposures over time (*3*). Single-base substitution (SBS) signatures are the indicators of mutational processes in cancer genomes that can be inferred through a computational decomposition of somatic single-nucleotide variants (SNVs) and their trinucleotide sequence context in large cancer genomics datasets (*4*, *5*). SBS signatures have been linked to clock-like mutational processes of aging (*6*), deficiencies in DNA repair pathways (*7*), endogenous mutational processes such as the activity of APOBEC cytidine deaminases (*8*), environmental carcinogens such as ultraviolet (UV) light (*9*), lifestyle exposures such as tobacco smoking (*10*), dietary components such as aristolochic acid (*11*), as well as the effects of cancer therapies (*12*, *13*). The causes of other signatures remain uncharacterized. Mutational signatures are increasingly found in healthy tissues, indicating that the mutational processes are active in normal and precancerous cells (*14*, *15*). Specific driver mutations in cancer genomes have been attributed to certain mutational processes (*16*, *17*). While some mutational signatures identified in cancer genomes can be reproduced in experimental systems (*9*, *18*, *19*), their mechanistic and etiological characterization is an ongoing challenge. As mutational processes are thought to predominantly generate passenger mutations, their broad functional implications on protein function and cellular pathways remain incompletely understood.

Here, we hypothesized that the mutational processes of SNVs specifically affect protein-coding sequence because of their trinucleotide sequence preferences encoded in SBS signatures. By characterizing the co-occurrence of mutational signatures and the sequence impact of associated SNVs in thousands of cancer genomes, we find that nonsense SNVs corresponding to stop-gain mutations (SGMs) are strongly associated with the mutational processes of tobacco smoking, APOBEC, and reactive oxygen species (ROS). SGMs are the most impactful class of SNVs that cause premature stop codons and result in truncated proteins or nonsense-mediated decay. The consequences of these mutational processes appear as driver mutations in tumor suppressor genes (TSGs) and hallmark cancer pathways. These processes represent preventable carcinogenic exposures as well as endogenous sources of DNA damage, and their association with premature stop codons is explained by their sequence-specific interactions with the genetic code. Our report provides direct evidence of the functional genetic impact of mutational signatures in cancer genomes and their interactions with the molecular and lifestyle drivers of the mutational processes, suggesting a role for SGM signatures in tumor heterogeneity and progression.

## RESULTS

### SGMs in tobacco smoking, APOBEC, and ROS signatures in cancer genomes

**Fig. 1.**
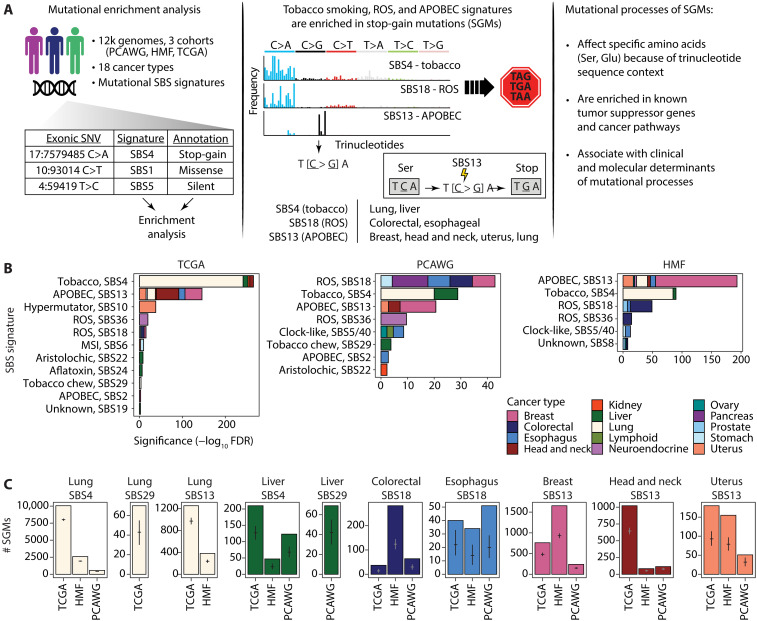
Protein-coding impact of mutational signatures in cancer genomes and associations with SGMs. (**A**) Overview of the study. Left: The associations of protein-coding impact of somatic SNVs and mutational signatures of SBSs were studied using enrichment analysis in >12,000 cancer genomes. Middle: SGMs were enriched in the SBS signatures of tobacco smoking, APOBEC, and ROS. These are explained by the trinucleotide preferences of the mutational processes that affect certain amino acid codons and convert these to stop codons. Right: Mutational signatures of SGMs were further studied in the context of driver genes and pathways, as well as the clinical and molecular correlates of the mutational processes. (**B**) Enrichments of mutational signatures in SGMs in multiple cancer types and in the three cancer genomics datasets (TCGA, HMF, and PCAWG) (FDR < 0.01). Bar plots show the cumulative significance of enriched SBS signatures in SGMs in various types of cancer. Tobacco smoking, APOBEC activity, and ROS exposure are the major mutational processes that contribute SGMs in multiple cancer types. (**C**) Observed and expected counts of SGMs derived from the most significant mutational processes. Mean expected mutation counts with 95% confidence intervals (CI) from binomial sampling are shown on the bars.

To study the protein-coding impact of SBS signatures, we analyzed 12,341 cancer genomes from 18 major tissue sites using data in three pan-cancer cohorts: The Cancer Genome Atlas (TCGA) PanCanAtlas (*20*) with 6509 exomes, Pan-Cancer Analysis of Whole Genomes (*4*) (PCAWG) with 2360 whole genomes, and the Hartwig Medical Foundation (*21*) (HMF) with 3472 whole genomes (Fig. 1A and fig. S1). Hypermutated and low-confidence samples were excluded (Materials and Methods). A total of 1.75 million exonic SNVs were classified on the basis of their protein-coding function as missense (67.4%), silent (27.7%), stop-gain (4.6%), stop-loss (0.1%), and start-loss (0.1%) mutations. We used consensus mutational signature calls from PCAWG (*4*) and annotated the signatures in the TCGA and HMF datasets using the SigProfiler software (*5*). Using these three datasets allowed us to replicate our findings across sequencing platforms, variant calling pipelines, and signature analysis methods. We then performed a mutation enrichment analysis by asking which specific mutational signatures were found in the five functional SNV classes more often than expected from chance alone. Systematic analysis of the 18 cancer types in the three genomic datasets revealed 332 associations of mutational signatures and protein-coding variant function [Fisher’s exact test, false discovery rate (FDR) < 0.01] (fig. S2).

We focused on SGMs (i.e., nonsense SNVs), the most disruptive class of SNVs that induces protein truncations and loss of function (LOF). SGMs were consistently enriched in the SBS signatures of three major mutational processes of tobacco smoking, APOBEC activity, and ROS (Fig. 1, B and C). First, the tobacco smoking signature SBS4 with frequent C>A transversions (*22*) was enriched in SGMs in primary lung cancers in TCGA [10,054 versus 8006 expected SGMs, fold change (FC) = 1.26; FDR = 4.6 × 10^−242^; Fisher’s exact test] and metastatic lung cancers in HMF (FC = 1.34; FDR = 1.9 × 10^−85^). Similarly, SGMs were also enriched in the SBS4 signature in the three cohorts of liver cancer samples (FDR < 10^−5^). The SBS29 signature attributed to tobacco chewing was also associated with SGMs in lung and liver cancers (FDR < 0.001).

Second, the APOBEC signature SBS13 was enriched in SGMs in multiple cancer types, especially in breast (1653 SGMs observed versus 931 expected, FDR = 1.1 × 10^−138^; HMF), head and neck (FC = 1.58; FDR = 3.3 × 10^−53^; TCGA), uterine, lung, and esophageal cancers. Notably, SBS13 appeared as the predominant APOBEC signature of SGMs, while few or no enrichments were seen for the alternative APOBEC signature SBS2. SBS13 and SBS2 both preferably affect TCN trinucleotides; however, SBS13 is primarily characterized by C>G and C>A mutations, while C>T mutations are common to SBS2. These differences between the two signatures explain the preferential enrichment of SBS13 to convert codons with TCN trinucleotides to stop codons (TAG, TAA, and TGA).

Third, SBS18 and SBS36, the two mutational signatures associated with ROS, were also enriched in SGMs. These SBS signatures characterized by C>A mutations were especially enriched in cancers of the digestive system such as colorectal cancer (HMF: 282 SGMs observed versus 124 expected; FDR = 5.0 × 10^−37^), esophageal cancer and stomach cancer, as well as pancreatic, neuroendocrine, and breast cancers. As ROS signatures were overall less frequent in cancer genomes than other signatures, fewer SBS18-associated SGMs were also found. Less-frequent carcinogenic signatures of aflatoxin and aristolochic acid exposures were also associated with SGMs. These observations were consistent in primary and metastatic cancers, and their detection in independent whole-genome sequencing (WGS) and whole-exome sequencing (WES) datasets also lends confidence to our findings.

We validated the associations of SGMs and SBS signatures with additional analyses. First, we repeated the enrichment analysis using a probabilistic approach that accounted for all potential sample-specific signature exposures in annotating individual SNVs. By sampling these signature annotations repeatedly, we confirmed that SGMs remained highly enriched in the SBS signatures of tobacco smoking, APOBEC, and ROS (fig. S3). The probabilistic analysis also showed an even stronger enrichment of APOBEC signatures in SGMs in lung cancer compared to the analysis of top signature annotations, while the pronounced enrichment of SGMs in the smoking signature was somewhat attenuated in the probabilistic analysis. Previous studies indicate that both the tobacco smoking and the APOBEC processes contribute somatic mutations in lung cancer whereas APOBEC is more involved in later mutagenesis (*23*), potentially explaining this observation. Accordingly, a subset of SGMs were likely attributable to either tobacco smoking or APOBEC signatures, or the age-associated signature SBS5 (fig. S4). Second, we performed a pan-cancer analysis by combining samples of all cancer types and again recovered the tobacco smoking, APOBEC, and ROS signatures with very strong enrichments of SGMs (fig. S5). Thus, the exogenous mutational process of tobacco smoking and the endogenous processes of APOBEC activity and ROS appear as major drivers of disruptive protein-truncating mutations that may directly affect protein function and regulation in cancer.

We also reviewed the enriched SBS signatures of missense and silent SNVs (fig. S2). Silent mutations were consistently enriched in the mitotic clock-like signature SBS1 in most cancer types and the three cohorts. SBS1 includes C>T transitions caused by 5-methylcytosine deamination. In contrast, missense SNVs were often enriched in the common clock-like signatures SBS5 and SBS40 that have relatively featureless (flat) trinucleotide profiles. Silent and missense SNVs are broad categories of variants that cover various trinucleotides in the genetic code; therefore, these associations with common mutational signatures are expected. Functional subclasses of missense mutations should be considered in future analyses. Associations with APOBEC signatures were also identified in multiple cancer types: SBS2 was often enriched in silent SNVs, while SBS13 was enriched in missense SNVs. Sample sizes and signature exposures determine the statistical power of detecting associations of SBS signatures and SNV annotations in the various cohorts. Together, these results exemplify the complex landscape of functional impacts that mutational processes enact on the protein-coding genome.

### SGM signatures and the genetic code

**Fig. 2.**
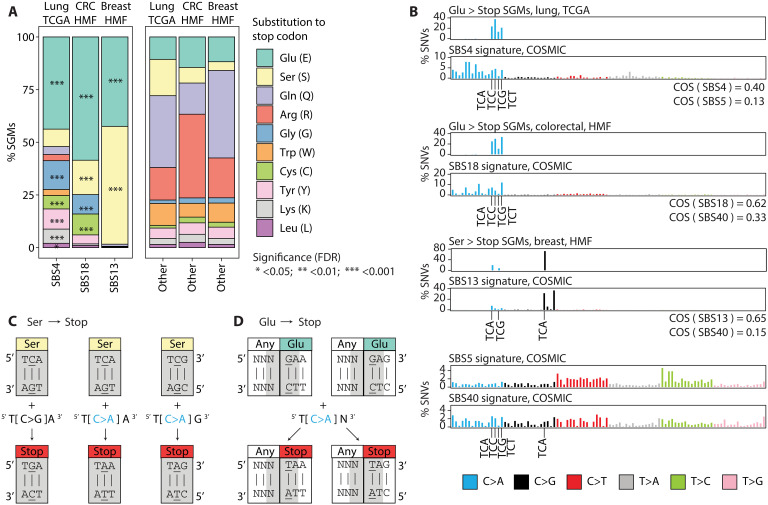
SBS signatures induce protein-truncating mutations by targeting the genetic code. (**A**) SGMs of tobacco smoking, APOBEC, and ROS signatures are enriched in specific amino acids. Bar plots show the proportions of amino acids affected by SGMs. Left: SGMs of the three major signatures: SBS4 in lung cancer, SBS18 in colorectal cancer, and SBS13 in breast cancer. Right: Control SGMs of all other mutational signatures in these cancer types. Enrichment of signature-associated SGMs relative to controls are shown as asterisks (Fisher’s exact tests). (**B**) Trinucleotide profiles of SGMs of glutamic acid and serine residues (Glu>Stop, Ser>Stop) and the reference COSMIC signatures for SBS4, SBS18, and SBS13. As controls, the profiles of the most frequent SBS signatures are shown (SBS5/40; two plots at the bottom). Cosine similarity (COS) scores comparing the SGM signatures and the COSMIC reference signatures are shown. The trinucleotide substitutions from (C) and (D) are highlighted on the *x* axis. (**C** and **D**) SBS signatures interact with the genetic code to induce stop codons. The codons encoding serines and glutamic acids are shown as rectangles. The trinucleotides inducing stop codons upon mutation are shown in gray. Base mutations are underlined. (C) Serine substitutions to stop codons. C>G and C>A transversions in SBS13 and SBS18 induce stop codons by substituting the middle nucleotides of serine codons (yellow). (D) Glutamic acid substitutions to stop codons. C>A transversions in SBS4, SBS13, and SBS18 induce stop codons by affecting two consecutive codons. Because SBS signatures are represented with pyrimidines as reference nucleotides, this schematic shows the reverse complements of the trinucleotides in which glutamic acids are changed to stop codons. Here, the reverse-complementary trinucleotide transversions characteristic of the tobacco smoking signature SBS4 substitute the first nucleotide in the glutamic acid codon (teal).

To explore the genetic mechanism underlying the enrichments of SGMs in the three major mutational processes, we studied the types of amino acids that were most frequently substituted by stop codons, focusing on tobacco smoking, APOBEC, and ROS signatures in lung, breast, and colorectal cancers. Several types of amino acids were unexpectedly frequently replaced by stop codons. Glutamic acid (Glu) residues showed the strongest enrichment of stop codon substitutions in all three SBS signatures (Fig. 2A). In lung cancer, Glu>Stop substitutions were enriched fourfold in the tobacco smoking signature SBS4 compared to other signatures (43.8% versus 10.6%, FDR < 10^−300^). Glutamic acid residues were also significantly affected by the APOBEC signature in breast cancer and ROS signatures in colorectal cancer (FC > 3.6; FDR < 1.7 × 10^−57^). As expected, these enrichments are also supported by the reference SBS signatures of the Catalogue of Somatic Mutations in Cancer (COSMIC) database (*24*) (Fig. 2B and fig. S6). On the basis of cosine similarity (COS) scores, the SNV trinucleotide profiles corresponding to Glu>Stop substitutions in our data were considerably more similar to the COSMIC reference SBS signature profiles of tobacco smoking and ROS (lung: COS_SBS4_ = 0.40; colorectal: COS_SBS18_ = 0.62), compared to the frequent clock-like signatures SBS5 and SBS40 that we used as controls (lung: COS_SBS5_ = 0.13; colorectal: COS_SBS40_ = 0.33). Besides Glu>Stop substitutions, other types of amino acids were also enriched in stop codon substitutions in tobacco smoking and ROS signatures, including glycines (13.7%) and cysteines (6.4%) (FC > 4.8; FDR < 3.6 × 10^−60^), while arginine, glutamine, and other residues were less frequently affected by SGMs than expected.

The APOBEC signature SGMs in breast cancer encoded stop codon substitutions almost exclusively in serine and glutamic acid residues (55.9 and 42.4%, respectively). Ser>Stop substitutions were more frequent in SBS13 compared to other signatures (4.2% expected; FC = 13, FDR < 10^−300^). Accordingly, the SNV trinucleotide profiles encoding Ser>Stop substitutions showed a considerably higher COS to the COSMIC reference APOBEC signature (COS_SBS13_ = 0.65) than the more common SBS40 reference signature we used as a control (COS_SBS40_ = 0.15), confirming the mutational signature annotations of SGMs in our data. Last, ROS signatures were also enriched in Ser>Stop substitutions in colorectal cancer (FDR = 6.2 × 10^−6^), while no enrichment of SGMs in serine residues was seen in tobacco smoking signatures in lung cancer.

To consolidate these statistical associations into a mechanistic model, we examined the genetic code of the most common stop codon substitutions involving serine and glutamic acid codons (Fig. 2, B and D). First, APOBEC-associated Ser>Stop substitutions in breast cancer were predominantly encoded by T[C>G]A transversions (76.8%) as well as T[C>A]A and T[C>A]G transversions. The TCA and TCG trinucleotides encode the two serine codons that require an SBS to become stop codons. The corresponding stop codons TGA, TAA, and TAG are induced by three of the transversions distinctive of the SBS13 APOBEC signature (Fig. 2C). Second, the Glu>Stop substitutions apparent in tobacco smoking and ROS signatures were predominantly caused by T[C>A]N transversions that overlap two adjacent codons (Fig. 2D). Here, the reverse-complementary trinucleotides NGA include the two first nucleotides of glutamic acid codons GAA and GAG, which are replaced with the stop codons TAA and TAG upon N[G>T]A transversions, respectively. Last, glutamine (Gln) and arginine (Arg) codons often seen in the SGMs of other SBS signatures are incompatible with the signatures SBS4, SBS13, and SBS18 (fig. S7). In summary, the genetic code explains the genesis of SGMs by the mutational processes of tobacco smoking, APOBEC, and ROS.

### Driver genes and pathways with truncating mutations

**Fig. 3.**
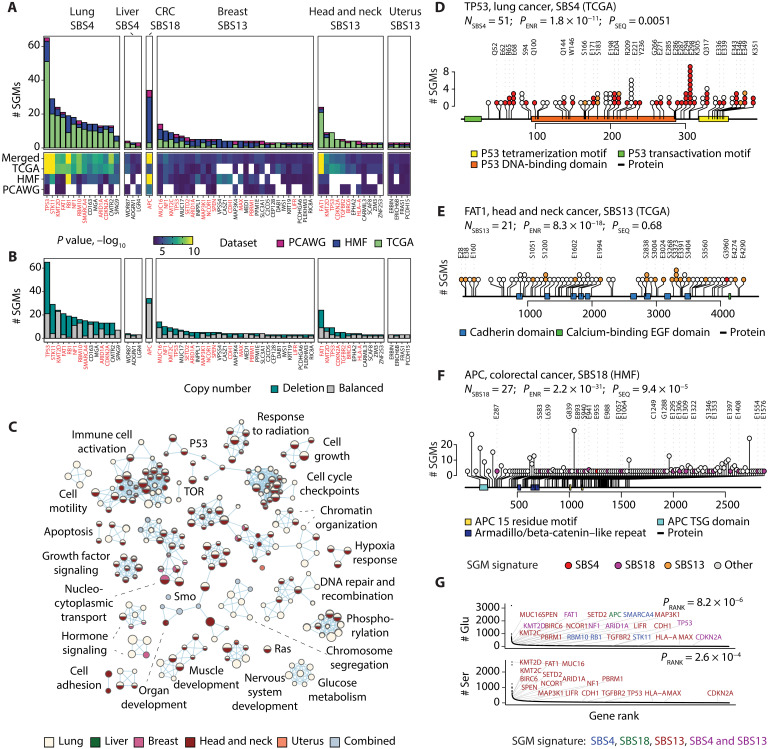
SGMs of tobacco smoking, APOBEC, and ROS signatures are enriched in TSGs and cancer hallmark pathways. (**A**) Genes enriched in SGMs of the signatures SBS4, SBS13, and SBS18. Each cancer type was analyzed separately, and the identified genes were integrated across the three cancer cohorts (Fisher’s exact tests, Brown FDR < 0.05). Known cancer genes are shown in red. (**B**) SGMs in the enriched genes often co-occur with genomic CN losses in the same cancer samples, indicative of loss of heterozygosity. (**C**) Biological processes and molecular pathways with enriched SGMs of SBS4 and SBS13. Significant pathways were identified by merging evidence through the SGM signatures and the cancer types (ActivePathways, FDR < 0.05). The enrichment map is a network of enriched pathways shown as nodes in which edges connect pathways with many shared genes. Nodes are colored by the cancer type in which SGM enrichment was detected. Light blue represents pathways that reached significance only after combining the evidence from the five cancer types. (**D** to **F**) Examples of TSGs enriched in SGMs of the three mutational signatures. SGMs in *TP53* in lung cancer, *FAT1* in head and neck cancer, and *APC* in colorectal cancer are enriched in SBS4, SBS13, and SBS18, respectively. Colored circles show the signature-associated SGMs, their reference amino acids, and their sequence positions. Pfam protein domains are shown as colored rectangles. The number of SBS-associated SGMs (*N*_SBS_), the enrichment *P* value of SGMs (*P*_ENR_, Fisher’s exact test), and the *P* value of SGMs accumulating toward either protein terminus (*P*_SEQ_, one-sample Wilcoxon rank sum test) are shown. EGF, epidermal growth factor. (**G**) Genes enriched with SBS4, SBS13, and SBS18 SGMs have a higher amino acid sequence content of Ser and Glu residues compared to all protein-coding genes (*P*_RANK_, Mann-Whitney *U* test). Colors indicate the mutational signatures enriched in SGMs.

To study the functional consequences of SGM signatures, we asked whether the mutations converge on specific genes. We focused on the six types of cancer in which consistent evidence of SGM signatures was found in the TCGA, PCAWG, and HMF datasets. We identified 56 genes with significantly enriched SGMs of tobacco smoking, APOBEC, and ROS signatures relative to the exome-wide distributions of these signatures and all SGMs: 14 genes in lung and liver cancers with the tobacco smoking signature SBS4; 44 genes in breast, head and neck, and uterine cancers with the APOBEC signature SBS13; and 1 gene (*APC*) with the ROS signature SBS18 in colorectal cancers (Fig. 3A and table S1) (Brown FDR < 0.05, Fisher’s exact tests). The genes included 556 signature-associated SGMs in 467 cancer genomes in the combined datasets, representing 3.8% of all tumors we studied. A large fraction of these (24 genes or 43%) are known cancer genes from the COSMIC Cancer Gene Census (CGC) database (*25*), more than expected from chance alone (24 found, 2 of 56 genes expected; *P* = 1.4 × 10^−20^). Several core TSGs such as *TP53*, *FAT1*, *CDH1*, *RB1*, *NF1*, and *APC* were identified.

To further interpret the mutations functionally, we prioritized the genes with SGMs across cancer types, excluding colorectal cancer for which only one gene was found. Integrative pathway enrichment analysis of SGM-ranked genes across the cancer types using the ActivePathways method (*26*) highlighted biological processes and pathways such as apoptosis, growth factor signaling, cell motility, and cell proliferation and development (FDR < 0.05; Fig. 3C). Most detected pathways were identified in multiple cancer types, primarily through the tobacco signature in lung cancer and the APOBEC signature in head and neck cancer. Thus, the SGMs generated by these mutational processes converge to TSGs and cancer pathways and thereby contribute to LOF, oncogenesis, and tumor progression.

Mutational signatures of tobacco smoking, APOBEC, and ROS were enriched in truncating mutations in important cancer genes. First, SGMs associated with tobacco smoking were enriched in 14 genes in lung cancer across the three datasets, including *TP53*, in which most SGMs in the TCGA cohort (51 of 95) were driven by SBS4 (15 SBS4 SGMs expected, FDR = 1.8 × 10^−11^) (Fig. 3D). Truncations in *TP53* preferentially occurred toward the disordered C-terminal tail involved in protein tetramerization (*P* = 0.0051, one-sample Wilcoxon rank sum test) in which SGMs have been associated with LOF phenotypes of TP53 (*27*) and where posttranslational modification sites are often mutated in cancer (*28*). SGMs of SBS4 and SBS13 showed high levels of functional activity in saturation mutagenesis screens of TP53 (*27*), supporting their roles in cancer phenotypes (fig. S8). In head and neck cancer, *TP53* included SGMs of SBS4 as well as SBS13, suggesting an interplay of the two mutational processes (fig. S9). A few SBS4 SGMs in *TP53* were also identified in lung cancers of patients with no smoking history in TCGA (fig. S9). As another example, the second-ranking gene *STK11* had 20 of 22 SGMs attributable to the tobacco smoking signature in the TCGA dataset (1 SBS4 SGM expected; FDR = 5.6 × 10^−17^) (fig. S9). Inactivating mutations of the bona fide TSG *STK11* (i.e., LKB1; serine/threonine kinase 11) are common in lung cancer, modulate differentiation and metastasis in vivo, and have been observed more frequently patients with smoking history (*29*–*31*). Smoking-associated truncations in STK11 accumulated toward the N terminus of the protein (*P* = 0.002), suggesting that the mutational process of tobacco smoking directly contributes to early truncations and LOF of this TSG. Similar enrichments of SGMs were seen in other core TSGs such as *RB1*, *NF1*, and *ARID1A* and emerging TSGs such as *MGA*, a transcription factor of the MYC network that suppresses growth and invasion in cellular and mouse models of lung cancer (*32*).

Second, SGMs of the APOBEC signature SBS13 were enriched in 44 genes in breast, head and neck, and uterine cancers. The most significant gene, *FAT1*, was found in head and neck cancer in TCGA and included 21 APOBEC-associated SGMs (1 SBS13 SGM expected, *P* = 8.3 × 10^−18^) (Fig. 3E). *FAT1* encodes a proto-cadherin and master regulator of the Hippo pathway that controls organ growth, cell polarization, and cell-cell contacts. *FAT1* is one of the most frequently mutated TSGs in cancer whose LOF enhances tumor invasiveness, metastasis, and drug resistance (*33*, *34*), suggesting a link between APOBEC-induced protein truncations and disease outcomes. *FAT1* was also found in lung cancer where SGMs were enriched in the tobacco signature SBS4. Besides *FAT1*, SGMs of the APOBEC signature were also seen in other hallmark cancer genes such *CDH1*, *TP53*, *CDKN2A*, and *TGFBR2*, and putative cancer genes such as the receptor tyrosine kinase *EPHA2* that regulates glutamine metabolism in cancer through the Hippo pathway (*35*).

Third, the ROS signature SBS18 was enriched in SGMs in one gene, *APC*, which was identified in metastatic colorectal cancers of the HMF cohort (27 of 346 SGMs versus 1 expected; *P* = 2.2 × 10^−31^) (Fig. 3F). APC inactivation is an early oncogenic event that disrupts β-catenin degradation and activates Wnt signaling (*36*, *37*). This suggests a link of *APC* loss with the oxidative stress in the tumor microenvironment or diet or with the therapies of metastatic cancers (*38*).

TSGs are often inactivated in cancer through multiple mechanisms. To determine whether TSGs were inactivated in samples with signature-associated SGMs, we asked whether the 56 SGM-enriched genes were also altered by genomic copy number (CN) losses in the same tumor samples (Fig. 3B). The SGM-enriched genes appeared to carry both SGMs and CN losses in most relevant cancer samples (58.9% or 275 of 467). This was also apparent in individual TSGs such as *TP53* (67.1% or 55 of 82 samples), *STK11* (86.2% or 25 of 29), and *FAT1* (73.3% or 33 of 45). Thus, some SGMs contributed by the mutational processes of tobacco and APOBEC drive biallelic inactivation of TSGs where one gene copy is inactivated by SGMs while the other copy is deleted.

We asked whether the SGM-enriched genes complied with our model of SGM signatures and the genetic code (Fig. 2, C and D). As expected, the proteins encoded by the 56 SGM-enriched genes contained significantly more glutamic acids relative to the reference human proteome (Wilcoxon rank sum *P* = 8.2 × 10^−6^) (Fig. 3G). Proteins with APOBEC-associated truncations also associated with a higher serine content (*P* < 2.6 × 10^−4^). The genetic model was also supported at the level of individual genes. For example, most SGMs of the tobacco smoking signature in *TP53* affected glutamic acids (32 of 51), while the APOBEC-associated SGMs in *FAT1* affected either serines (10 of 21) or glutamic acids (11 of 21). Furthermore, the same subset of core TSGs was enriched in both tobacco smoking– and APOBEC-associated SGMs in different cancer types (e.g., *TP53*, *CDKN2A*, *NF1*, *FAT1*, and *ARID1A*), as the SGMs introduced through the two mutational processes converge across different cancer types. Thus, certain TSGs may be more exposed to these SGM-inducing mutational processes because of their protein sequence content, indicating an interplay between the mutational processes and positive selection against tumor-suppressive function.

### Clinical and molecular associations of SGM signatures

**Fig. 4.**
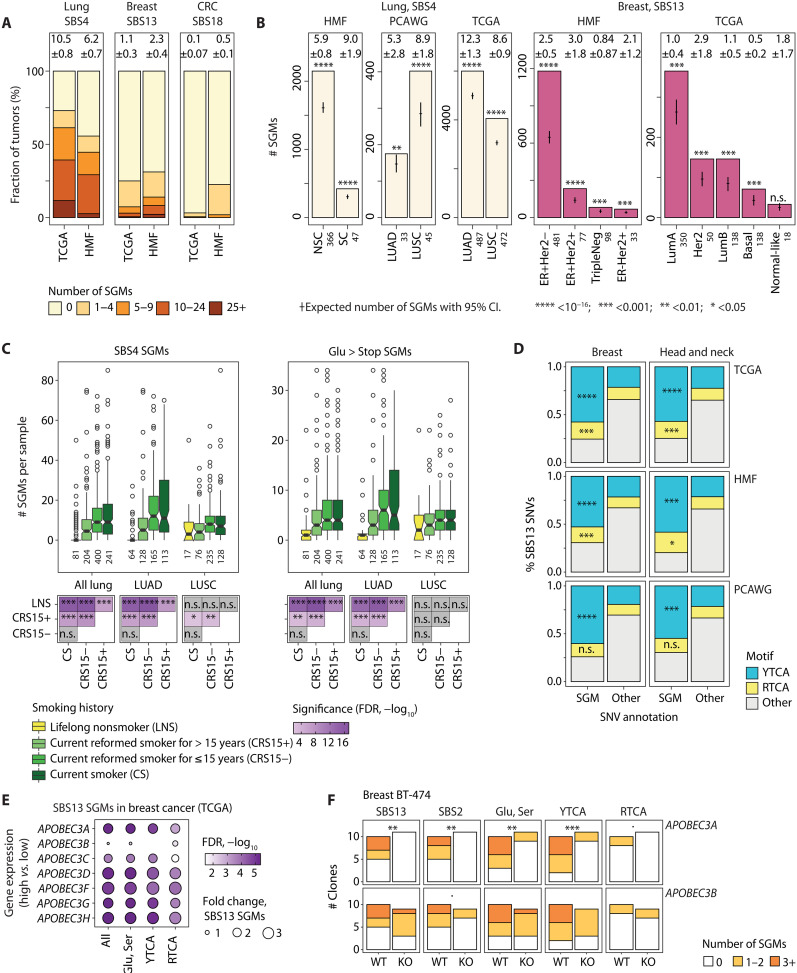
Molecular and clinical associations of SGMs with tobacco smoking and APOBEC activity. (**A**) Mutational burden of SGMs of SBS4, SBS13, and SBS18 in cancer genomes. Bar plots show SGM burden for these signatures in primary (TCGA) and metastatic cancers (HMF). Mean numbers of SGMs per cancer genome with ±95% CI are shown above the bars. (**B**) SBS4 and SBS13 SGMs are enriched in the molecular subtypes of lung and breast cancer. Expected total SGM counts with 95% CI are shown as points and whiskers. Sample sizes are shown on the *x* axis. Lung cancer subtypes include small cell (SC), non–small cell (NSC), adenocarcinoma (LUAD), and squamous cell carcinoma (LUSC). (**C**) SBS4 SGMs associate with the smoking history of patients with lung cancer in TCGA. Top: Box plots show the number of SBS4 SGMs (left) and Glu>Stop substitutions per cancer genome in patients grouped by smoking history (right), with sample sizes for each group shown in the *x*-axis labels. Bottom: Tile plot shows the statistical significance of the associations of smoking history and SGM burden (Wilcoxon rank sum test, FDR-adjusted). (**D**) SBS13 SGMs in breast and head and neck cancers are enriched in the DNA motif YTCA compared to other non-SGM SBS13 mutations (right). Fisher’s exact *P* values are shown. (**E**) *APOBEC3* gene expression in breast cancer associates with more frequent SBS13 SGMs. TCGA breast cancer samples were compared using median dichotomization of each *APOBEC3* gene (i.e., high versus low). FDR-adjusted *P* values of Wilcoxon rank sum tests are shown. (**F**) Breast cancer cell line clones with *APOBEC3A* knockout show a significantly reduced SBS13 SGM burden compared to *APOBEC3A*-WT clones, while no significant changes are seen in cells with *APOBEC3B* knockout. WGS data were retrieved from (*43*). *P* values were computed using negative binomial regression. n.s., not significant.

We quantified the mutational processes of SGMs in individual cancer genomes. The largest number of SGMs was associated with the tobacco smoking signature in lung cancers. In TCGA, lung cancer samples included 10.5 tobacco smoking–associated SGMs per genome on average, whereas 73% of cancers had at least 1 and 39% had at least 10 such protein-truncating mutations. In primary breast cancers in TCGA, APOBEC processes were associated with an average of 1.1 SGMs and affected a quarter of samples. In metastatic breast cancers of the HMF dataset, APOBEC-driven SGM burden was higher (mean 2.3 SGMs per sample; 32% of samples), consistent with longer or higher levels of APOBEC activity in advanced cancers (*39*). ROS-associated SGMs, while less frequent in cancer genomes overall, were most pronounced in metastatic colorectal cancers in HMF, affecting 23% of samples with an average of 0.5 SGMs per genome (Fig. 4A). Therefore, a large fraction of cancer genomes has some SGMs and potential LOF alterations through these mutational processes.

We next studied the activity of SGM signatures at the level of cancer subtypes. The enrichment of SGMs in the tobacco smoking signature was detected in primary lung adenocarcinomas (LUAD) and squamous cell carcinomas (LUSC) in TCGA, as well as metastatic non–small cell and small cell cancers in the HMF dataset (Fig. 4B). APOBEC associations with SGMs in breast cancer were also confirmed in the major histological and molecular cancer subtypes and were also detected in primary breast cancers and metastatic cancers originating from the breast. Notably, the Her2-positive breast cancer subtype in TCGA had threefold more APOBEC-driven SGMs than all other subtypes combined (2.9 versus 0.95 SGMs per genome) (Fig. 4B), consistent with earlier studies showing higher APOBEC activity in that subtype (*8*). Thus, the mutational processes generating SGMs are active across lung and breast cancer subtypes and in primary and metastatic cancers.

We assessed SGMs in the context of the smoking history of patients with lung cancer in TCGA. Compared to patients with a smoking history, the cancer genomes of lifelong nonsmokers had fewer tobacco-associated SGMs of SBS4 (FDR < 10^−6^) and fewer Glu>Stop substitutions (FDR < 10^−9^). No significant differences in SBS4 SGM burden were found between current and recently reformed smokers (FDR = 0.93); however, both groups had significantly more SBS4 SGMs than lifelong nonsmokers and long-term reformed smokers (FDR < 10^−6^). Cancer subtype analysis confirmed the association between lifetime smoking and the burden of SBS4 SGMs in LUAD, while weaker signals were observed in LUSC (Fig. 4C). This is expected as the LUAD subtype is more common among nonsmokers than LUSC (*40*) (13.6 and 3.7% in TCGA, respectively) and the more varied composition of the LUAD cohort may contribute to the more pronounced association with SGMs. Therefore, SGMs in lung cancer genomes can be attributed to lifetime smoking activity, indicating a preventable cause of these impactful genetic aberrations.

We analyzed the functional characteristics of APOBEC-related SGMs of signature SBS13. First, an extended sequence context analysis of SGMs showed a strong enrichment of mutations in YTCA motifs compared to non-SGM SBS13 mutations (Fig. 4D). This was apparent in breast cancer and head and neck cancer and was confirmed in all three cohorts. Second, we associated SGM burden in breast cancer with the expression levels of *APOBEC3* genes using matched genomic and transcriptomic datasets in TCGA (Fig. 4E). Breast cancer samples with higher *APOBEC3A* expression had significantly more APOBEC-driven SGMs compared to cancers with lower expression (*P* = 1.3 × 10^−6^, Wilcoxon test) while weaker or subsignificant associations were seen with *APOBEC3B* expression. Differential expression of other *APOBEC3* genes also associated with SGM burden. These findings were partially replicated in the HMF cohort of metastatic cancers, although with limited significance and sample size (fig. S10). The mRNA associations and frequent YTCA motifs suggest that the SGMs of the SBS13 signature are mostly associated with mutagenesis by APOBEC3A rather than APOBEC3B, in agreement with previous studies (*41*, *42*). Last, we studied WGS data from cancer cell lines with *APOBEC3A* or *APOBEC3B* knockout (KO) experiments (*43*). We found that the clones of breast cancer cell line BT-474 showed significantly fewer SGMs upon *APOBEC3A* KO compared to control clones with wild-type (WT) *APOBEC3A*, while *APOBEC3B* knockdown led to no significant differences in SGM burden (Fig. 4F). Attenuated signals were seen in two additional cell lines (fig. S11). These associations of APOBEC mutagenesis of SGMs with *APOBEC3* gene expression and DNA motifs connect the mutational processes of SGMs in patient cancer genomes with the expected molecular pathways and functional evidence. Overall, the clinical and molecular associations of mutational processes and SGMs provide insights to tumor heterogeneity and patient outcomes and have potential implications for biomarker and therapy development.

## DISCUSSION

Our pan-cancer analysis shows that the mutational processes of tobacco smoking, APOBEC, and ROS are sources of SGMs in cancer genomes. The trinucleotide contexts of these mutational processes are biased toward substitutions of glutamic acid and serine codons to stop codons, explaining the strong statistical associations observed in many cancer types. In support of this mechanism, we present several lines of evidence. First, the tumor suppressors with the strongest enrichments of SGMs also have a high protein sequence content of these amino acids. Second, we can identify the mutational processes of SGMs in large cohorts of primary and metastatic cancers of various cancer types and in WGS and WES datasets. Third, the mutational burden of SGMs correlates with the molecular drivers of the mutational processes, including lifelong tobacco exposure of patients with lung cancer and the expression levels of *APOBEC3* genes.

Our analysis ties together the functional impact of mutational processes and positive selection in cancer genomes. The genes with the most frequent SGMs associated with the three mutational processes are clearly enriched in core TSGs, including early oncogenic drivers such as *APC*, later drivers of tumor progression and metastasis such as *CDH1*, as well as less-characterized cancer genes such as *EPHA2* and *MGA*. In these genes, SGMs likely promote cancer development and are under positive selection. The trinucleotide preferences of mutational processes reveal their proteo-genomic characteristics: For example, the TSGs with enriched SGMs also often encode for proteins with many glutamic acids and serines, suggesting that such protein sequence content is more vulnerable to truncating mutations and LOF.

The mutational processes that contribute SGMs are the major processes of somatic mutagenesis in many cancer types. Tobacco smoking appears as the strongest driver of SGMs in lung, head and neck, and esophagus cancers, which involve direct exposure to smoke. We also find SGM enrichments in other carcinogenic processes of tobacco chewing, and the dietary carcinogens aflatoxin and aristolochic acids, that are detected less frequently in these cancer cohorts. Further, increased smoking is associated with a higher SGM burden, indicating that the more an individual is exposed to tobacco smoke, the more likely they are to acquire SGMs and disrupt gene function in tobacco-exposed cells. Therefore, loss-of-function mutations in cancer genomes appear to be influenced by lifestyle and environment factors, such as tobacco smoking or passive exposure to second-hand smoke.

APOBEC enzymes are core components of the innate immune system that are involved in antiviral defense and somatic antibody diversification (*44*). Aberrant APOBEC activity is a major cause of somatic mutations in many cancer types. APOBEC enzymes were described in virus restriction pathways that disable viruses by inducing missense and SGMs in viral RNA (*45*). This evolutionarily important mutational process of defense against pathogens corroborates our observations in cancer genomes. RNA editing by APOBEC1 causes a protein truncation in the apolipoprotein APOB that is required for lipid processing in the intestine (*46*, *47*). Somatic mutagenesis in normal human small intestine has been associated with APOBEC1 activity and includes SGMs in TSGs (*48*). At the mesoscale of ~30 bps, APOBEC3 affects DNA stem-loop structures and causes recurrent passenger mutation hotspots, while APOBEC3-driven mutations in cancer driver genes often lack these mesoscale features (*49*). At the regional scale, APOBEC3 mutagenesis is more pronounced in tissue-specific open-chromatin regions (*50*, *51*), indicating that the SGMs preferentially occur in actively expressed genes in which LOF mutations are more likely to have functional consequences. Last, mutational processes that generate SGMs and cause increased tumor heterogeneity may be reined in through therapeutic APOBEC3 inhibition (*52*), especially as APOBEC3 activity later in tumor evolution has been linked to subclonal diversification, driver mutations, and resistance to targeted therapy (*39*, *53*, *54**)*.

Oxidative stress and ROS are major sources of genomic instability that are associated with lifestyle factors common in developed countries, such as malnutrition, limited dietary antioxidant levels, obesity, and excessive alcohol consumption (*55*). Oxidative stress is also associated with some anticancer therapies such as ionizing radiation and certain chemotherapeutic agents. SGMs of ROS signatures were especially apparent in metastatic colorectal cancers that are commonly treated with radiation therapy. Rare cancer-predisposing germline mutations of the DNA repair enzyme MUTYH have been associated with the ROS signature SBS18 and more frequent SGMs (*56*), supporting our findings of SBS18-driven SGMs in cancer genomes. Thus, lifestyle variables, genetic makeup of patients, and certain cancer treatments may also contribute to loss-of-function mutagenesis.

As cancers evolve, they become more heterogeneous and their paths to progression and metastasis become more varied. This heterogeneity is likely acquired through additional mutations that further deregulate cancer pathways and unlock resistance to therapy. By inducing SGMs, the mutational processes of tobacco, APOBEC, and ROS directly contribute to tumor heterogeneity by causing LOF. While not all these SGMs occur in core TSGs and directly drive cancer phenotypes, SGMs may involve genetic interactions with the core driver genes. Synergistic interactions may provide additional context-specific advantages to tumors in cases where the SGMs disrupt protective mechanisms and thereby enhance the phenotypes caused by core driver genes. On the other hand, SGMs may lead to synthetic lethal interactions in which SGMs disrupt a pathway that the core oncogenic pathway depends on. These interactions may be exploited for therapy by targeting other components of the SGM-disrupted pathway. Deeper analyses of the proteogenomic impact of mutational processes and their etiology and genetic and lifestyle associations may lead to innovative biomarkers, mechanistic insights to cancer pathways, and leads for therapy development.

## MATERIALS AND METHODS

### Somatic mutations in cancer genomes

Previously published genomics datasets were used. We analyzed somatic SNVs in three cohorts of multiple cancer types: International Cancer Genome Consortium (ICGC)/TCGA PCAWG (*2*) with WGS data of primary cancers, HMF (*21*) with WGS data of metastatic cancers, and the TCGA PanCanAtlas (*20*) project with WES data of primary cancers. DNA sequencing of cancer tissues of human individuals was performed by ICGC, TCGA, and HMF consortia members outside of this study under a series of locally approved Institutional Review Board protocols. Informed consent was obtained from all human participants as part of previous studies. Ethical review of the current data analysis project was granted by the University of Toronto Research Ethics Board under protocol no. 37521. For TCGA PanCanAtlas data, we used the Multi-Center Mutation Calling in Multiple Cancers (MC3) dataset of TCGA variant calls (*57*). We removed hypermutated tumor samples defined as those with >90,000 SNVs in WGS data and with >1800 SNVs in WES data, corresponding to genomes with approximately >30 SNVs/Mbps (*n* = 69 for PCAWG; *n* = 306 for HMF; *n* = 806 for TCGA). We excluded SNVs that did not pass the MC3 quality filter in TCGA. In WES data, we also removed lower-confidence samples with very few mutations for increased confidence in signature decomposition (<20 SNVs; 977 samples in TCGA). In HMF data, we removed 140 duplicate cancer genomes of tumors of the same patients by selecting the sample with the highest tumor purity. We also removed 25 samples lacking HMF patient IDs. To enable analyses across the TCGA, PCAWG, and HMF cohorts, cancer types were consolidated to 18 meta-types based on organs and/or anatomical sites, with each cancer type including at least 25 samples in the three cohorts (fig. S1). In HMF, the organ of the primary tumor was used for cancer type classification. Cancers of less-frequent primary sites and of unknown origin (HMF) were excluded. In total, we analyzed 1,751,110 exonic SNVs. The functional effects of SNVs on protein-coding genes were annotated using the ANNOVAR software (*58*) (version 24 October 2019) by using the canonical protein isoforms of the genes. The final dataset contained 12,341 cancer genomes (2360 in PCAWG, 3472 in HMF, and 6509 in TCGA). This included some samples that were present in both the PCAWG and TCGA cohorts (*n* = 484). The duplicate samples between PCAWG and TCGA were retained to provide additional technical validation across the sequencing platforms, variant calling pipelines, and signature-mapping strategies used to produce the datasets.

### SBS signatures

Mutational signatures for SBSs in PCAWG were retrieved from the consensus PCAWG dataset (*4*). In HMF and TCGA datasets, we separately assigned known SBS signatures to SNVs using the SigProfilerSingleSample software (version 0.0.0.27) (*5*) and the COSMIC SBS signature catalog (version 3) (*5*, *24*). For most analyses, each SNV was assigned to the most probable SBS signature based on these signature exposure predictions. We removed a small subset of samples in WGS data that were potentially contaminated with sequencing artifacts as defined by the presence of more than 20% of SNVs assigned to SBS27, SBS43, and SBS45 to SBS60, comprising nine samples in PCAWG and four samples in HMF. The TCGA dataset was not further filtered beyond the MC3 quality filter (*57*). To verify the tobacco, APOBEC, and ROS signatures of SGMs in lung, breast, and colorectal cancers, respectively, we computed COS scores to evaluate the similarity of the SGM trinucleotide profiles with the reference profiles of SBS trinucleotide frequencies in the COSMIC database. COS scores were computed separately for all SGMs and for Ser>Stop and Glu>Stop SGMs. As controls, we also computed the equivalent COS scores comparing the SGM trinucleotide profiles with the two clock-like featureless SBS signatures, SBS5 and SBS40, which were the most common SBS signatures in the respective cancer types.

### Enrichment analysis of protein-coding SNV classes and mutational signatures

We performed a comprehensive enrichment analysis of functional SNV annotations and mutational SBS signatures by separately comparing all consolidated cancer types in the three cancer cohorts. The analysis evaluated whether the classes of exonic SNVs [i.e., missense, stop-gain (i.e., nonsense), silent, start-loss, and stop-loss] were enriched in certain mutational signatures by co-occurring more often than expected from the independent distributions of these SNV classes and the SBS signatures in all protein-coding regions of a given cancer type and cohort. For each cancer type, we tested the signatures that were reasonably frequently detected, had at least 100 SNVs per cancer type and cohort, and included at least 1 SNV of the tested variant annotation class (e.g., SGM), excluding signatures annotated as sequencing artifacts in the COSMIC database (see above). Certain signatures associated with the common mutational processes were combined: clock-like signatures SBS5 and SBS40 (SBS5/40), UV signatures SBS 7a/b/c/d (SBS7), hypermutation-associated signatures SBS10a/b (SBS10), and the signatures SBS17a/b (SBS17). Because this analysis focused only on protein-coding regions, and to provide comparable analyses of WGS and WES datasets and reduce the inflation of significance in better-powered WGS datasets, we excluded nonexonic variants from the statistical tests. Statistical analysis was conducted using one-tailed Fisher’s exact tests that asked whether a set of SNVs derived from a given SBS signature and another set of SNVs with a given functional annotation overlapped more often than expected by chance alone. The resulting *P* values were adjusted for multiple testing using the Benjamini-Hochberg FDR method (*59*). Results were considered significant if FDR < 0.01. Expected values of mutations sharing SBS signatures and functional annotations were sampled from the independent binomial distributions over 10,000 iterations, parametrized by the product of the probabilities of signature mutations and functional annotations, respectively. Using a similar approach, we also asked whether specific types of amino acids were more likely to be substituted with stop codons through the SGMs driven by the identified SBS signatures. This analysis focused on only three cancer types and three SBS signatures in the cohorts with the strongest signals (SBS4 in lung cancer in TCGA, SBS13 in breast cancer in HMF, and SBS18 in colorectal cancer in HMF). Fisher’s exact tests were performed to assess whether certain amino acids co-occurred with the signatures more often than expected from the individual distributions of the signature-associated variants and the substituted amino acids. The resulting *P* values were corrected for multiple testing using FDR.

### Confirming the enrichment of SGMs in the major SBS signatures with probabilistic sampling

All major analyses in our study considered the most probable SBS signature for each SNV. To confirm our findings by accounting for the uncertainty in the signature annotations of individual SNVs and tumor samples, we performed a sampling analysis in which we assigned signatures to individual SNVs probabilistically over 100 iterations. Each SNV was assigned an SBS signature based on the multinomial distribution parametrized by the probabilities of all the SBS signatures identified in the given cancer genome. This procedure allowed the less-probable signatures to be included in the SNV annotation on the basis of their probabilities. The 100 probabilistically sampled SNV-to-signature assignments were then analyzed using the enrichment analysis approach described above, to determine which signatures were enriched in SGMs in various cancer types. Results were adjusted for multiple testing separately in each iteration and labeled on the basis of significance (FDR < 0.05). The FCs and FDR values of the SGM enrichments in different iterations were visualized as volcano plots.

### Analysis of SBS signatures and SGMs in genes

Genes with significant signature-associated SGMs were identified using one-tailed Fisher’s exact tests separately for the three major signatures (SBS4, SBS13, and SBS18). The tests compared the distribution of SGMs of each SBS signature in a gene relative to the distributions of all SGMs and all mutations of that SBS signature in all protein-coding genes combined. This analysis only used exonic mutations and excluded mutations in noncoding regions, similarly to the exome-wide analysis described above. Fisher’s exact tests were conducted for each gene separately and in all three cohorts separately (TCGA, HMF, and PCAWG). Genes were only tested if they had at least one SGM assigned to the given mutational signature. The resulting *P* values for each gene were merged across datasets using the Brown method (*60*) and corrected for multiple testing using FDR. Significant genes were selected on the basis of the Brown merged FDR values (FDR < 0.05). Known cancer genes from the COSMIC CGC database (*25*) (version 17 September 2020, accessed 21 October 2021) were highlighted in the resulting gene list. A Fisher’s exact test was used to determine whether the CGC genes were found in the list more often than expected, using all protein-coding genes as the background set. In an additional analysis, all protein-coding genes were ranked according to the numbers of glutamic acid (Glu) and serine (Ser) residues in their canonical protein isoforms. Genes identified in the SGM enrichment analysis from above were tested for higher-than-expected Glu or Ser content using one-tailed Mann-Whitney *U* tests relative to all canonical human proteins as reference. Also, for each candidate gene, we determined whether the sequence positions of the signature-associated SGMs were distributed toward either the N or C terminus of the protein more often than expected. One-tailed one-sample Wilcoxon rank sum tests were used for this analysis. Mutations in protein sequence were visualized using the lolliplot method of the trackViewer R package (*61*), using protein domain information retrieved from the Pfam database (*62*).

### Functional impact of SGMs in *TP53*

To analyze the functional impact of signature-associated SGMs in *TP53*, we obtained data from saturation mutagenesis screens from the study by Giacomelli *et al.* (*27*) and compared the *z* scores of TP53 functional activity among three classes of SNVs: (i) SGMs associated with SBS4 and/or SBS13 in any cancer sample in our datasets (i.e., PCAWG, TCGA, and HMF combined), and as controls (ii) missense SNVs observed in any cancer sample in our datasets, and (iii) all other mutations of *TP53* studied in the mutagenesis screens but not found in human cancer genomes. Only unique mutations were analyzed. Statistical significance between the groups was determined using Wilcoxon rank sum tests.

### Analysis of copy number alterations and SGMs

We aimed to identify potential biallelic inactivation cases where the gene was disrupted by both SGMs and CN alterations leading to genomic losses of the gene in the same tumor. We studied the 56 genes with enriched signature-associated SGMs from our analysis that included 556 SGMs in 467 tumors in total. Separate strategies to select copy number alterations (CNAs) were used for the TCGA dataset and the PCAWG and HMF datasets. For TCGA samples profiled previously using SNP6 microarrays, we analyzed the relative digital somatic CN calls of each gene as from previous consensus datasets. Gene losses in TCGA were defined through gene CN < 0.0. For PCAWG and HMF samples previously profiled using WGS, we analyzed the CN values of genomic segments defined in these projects. To define the CN value for each gene, we considered the overlapping genomic segment with the lowest CN and of at least 1000 bps in length. To define gene losses in PCAWG and HMF, we used different criteria for autosomes and the X chromosome and for samples with and without potential whole-genome duplication (WGD) events. A cancer genome was predicted to have undergone WGD if the genome-wide CN > 2.5. For non-WGD samples, we defined gene losses in autosomes through gene CN < 1.5. For WGD samples, we defined gene losses through gene CN < 2.0. The same thresholds were used to define gene losses in X chromosomes in female patients. Gene losses in X chromosomes in males were defined through gene CN < 1.0 for non-WGD samples and through gene CN < 1.5 for WGD samples. CNAs were unavailable for one relevant HMF sample and nine relevant TCGA samples, for which we assumed that no gene deletion events occurred.

### Pathway enrichment analysis

To understand the functional importance of the genes with SGMs of different mutational signatures, we performed an integrative pathway enrichment analysis using the ActivePathways method (*26*). The analysis was designed to prioritize genes and pathways that were enriched with signature-associated SGMs in multiple cancer types. We included the cancer types for which these genes were found, excluding colorectal cancer for which only one gene was found. For each cancer type, we selected the cohort with most cancer samples: lung (SBS4, TCGA), liver (SBS4, TCGA), breast (SBS13, HMF), head and neck (SBS13, TCGA), and uterine cancers (SBS13, TCGA). As the input to ActivePathways, we used a matrix of *P* values of all protein-coding genes and the selected cancer types, such that each *P* value reflected the enrichment of signature-associated SGMs in the gene and the cancer type. Gene sets of biological processes of Gene Ontology and molecular pathways of Reactome were derived from the Gene Matrix Transposed (GMT) files in the g:Profiler web server (*63*) (downloaded 3 January 2022). Gene sets with 100 to 500 genes were analyzed. Statistically significant pathways were selected (ActivePathways, FDR < 0.05). The results were visualized as an enrichment map (*64*), and the subnetworks were labeled interactively to find common functional themes of similar pathways and processes.

### Analysis of SGMs in tumor subtypes and correlation with patient smoking history

We studied the number of signature-associated SGMs in each cancer genome in the representative cancer types (SBS4 in lung, SBS13 in breast, and SBS18 in colorectal), and compared primary cancers in TCGA and metastatic cancers in HMF. Mean numbers of signature-associated SGMs per cancer genome were reported with 95% confidence intervals, by also including the samples where these SBS signatures were not detected. We also compared the per-tumor SGM counts separately in various subtypes of lung and breast cancer. Subtype analysis was not performed in the colorectal cancer cohort as subtype annotations were not available. Cancer subtype annotations for PCAWG were retrieved from the ICGC data portal, from patient information tables for HMF, and for TCGA from the TCGAbiolinks R package (*65*) (v. 2.18.0). Samples with unknown and missing subtype annotations were excluded. To validate the associations of SGMs and SBS signatures in cancer subtypes, we repeated the signature enrichment analysis of SGMs on relevant subsets of cancer samples using Fisher’s exact tests as described above. We also analyzed SGMs of the tobacco signature SBS4 in the context of smoking history of patients with lung cancer. We compared the subsets of TCGA cancer samples on the basis of the four categories of patient smoking history that were derived from TCGAbiolinks. We compared two categories of SGMs: SGMs assigned to SBS4 and SGMs causing Glu>Stop substitutions. Nonparametric Wilcoxon rank sum tests were used to compare mutation counts per patient in the four categories of smoking history. We performed one analysis by combining all patients with lung cancer on the basis of their smoking history, and two additional analyses focused on the two major histological subtypes (adenocarcinoma and squamous cell carcinoma).

### Motif analysis and correlation of *APOBEC3* expression with SBS13 SGMs

We examined the association between quadnucleotide DNA motifs and SGMs in breast cancer and head and neck cancer, in which the highest enrichments for SBS13 SGMs were found. We obtained the quadnucleotide sequence motifs for each variant and labeled them as YTCA (where Y is either C or T), RTCA (where R is either A or G), or as other (i.e., all other sequences). Fisher’s exact tests were then performed to determine whether SBS13 SGMs were more frequent at each motif type compared to non-SGMs of SBS13 as reference. Next, we associated the frequency of SGMs per cancer genome with the gene expression levels of APOBEC3 enzymes in breast cancer samples (*APOBEC3A*, *APOBEC3B*, *APOBEC3C*, *APOBEC3D*, *APOBEC3F*, *APOBEC3G*, and *APOBEC3H*). We analyzed breast cancer genomics datasets in TCGA and HMF using matching RNA sequencing (RNA-seq) datasets of the two cohorts. Cancer samples with no SBS13 mutations were also included in the analyses. We excluded cancer samples with no matching RNA-seq data. Each *APOBEC3* gene was analyzed separately. Samples were split (i.e., median-dichotomized) into two subsets on the basis of median *APOBEC3* gene expression. The resulting two groups for each *APOBEC3* gene were compared using one-tailed Wilcoxon rank sum tests to compare mutation counts per cancer genome. Four types of mutations were considered: all SGMs of the SBS13 signature, SGMs involving glutamic acids and serines combined (Glu>Stop, Ser>Stop), SBS13 SGMs with a YTCA motif, and SBS13 SGMs with an RTCA motif.

### Analysis of SGMs in cell lines from *APOBEC3* KO experiments

SNVs from five cell lines (breast: BT-474 and MDA-MB-453; B cell lymphoma: BC-1 and JSC-1; and bladder: HT-1376) in WT and *APOBEC3A* or *APOBEC3B* KO treatment groups were retrieved from the study by Petljak *et al.* (*43*). Clones considered nonunique by the authors were removed, and only daughter clones were used in this analysis. The functional effects of SNVs on protein-coding genes were annotated using ANNOVAR (version 24 October 2019), and SBS signatures were assigned to SNVs using SigProfiler as described above. *APOBEC3A* and *APOBEC3B* KO data were analyzed separately. Within each cell line and treatment group (KO or WT), SGMs in the following categories were counted: total SGMs, SBS13 SGMs, SBS2 SGMs, SGMs with a YTCA motif, and SGMs with an RTCA motif. Negative binomial regression models were fit on these counts using the treatment group as a covariate in the alternative model, and a two-model analysis of variance (ANOVA) with a chi-square test was used to determine statistical significance.
