# Supplementary material for: Mutational processes of tobacco smoking and APOBEC activity generate protein-truncating mutations in cancer genomes
Source: Sci Adv. 2023 Nov 3;9(44):eadh3083. doi: 10.1126/sciadv.adh3083 (PMC10624356; doi:10.1126/sciadv.adh3083)
Supplement: Supplementary file 1 — Figs S1 to S11 Legend for table S1 [file sciadv.adh3083_sm.pdf]

Science Advances
AAAS

Supplementary Materials for

Mutational processes of tobacco smoking and APOBEC activity generate protein-truncating mutations in cancer genomes

Nina Adler *et al.*

Corresponding author: Jüri Reimand, juri.reimand@utoronto.ca

Sci. Adv. **9**, eadh3083 (2023)
DOI: 10.1126/sciadv.adh3083

The PDF file includes:

Figs. S1 to S11
Legend for table S1

Other Supplementary Material for this manuscript includes the following:

Table S1

Combined cancer type	n total	PCAWG n	PCAWG subtype	PCAWG ST n	HMF n	HMF subtype	HMF ST n	TCGA n	TCGA subtype	TCGA ST n
**Biliary**	140	33	Biliary-AdenoCA	33	76	Biliary	76	31	Cholangiocarcinoma	31
**Bone**	512	94	Bone-Cart<br>Bone-Epith<br>Bone-Leiomyo<br>Bone-Osteosarc	9<br>11<br>34<br>40	209	Bone/Soft tissue	209	209	Sarcoma	209
**Breast**	1636	211	Breast-AdenoCa<br>Breast-DCIS<br>Breast-LobularCa	195<br>3<br>13	729	Breast	729	696	Breast invasive carcinoma	696
**Colorectal**	909	42	ColoRect-AdenoCA	42	522	Colon/ Rectum	522	345	Colon adenocarcinoma<br>Rectum adenocarcinoma	258<br>87
**Esophagus**	408	95	Eso-AdenoCa	95	135	Esophagus	135	178	Esophageal carcinoma	178
**Head & neck**	611	54	Head-SCC	54	60	Head & neck	60	497	Head and neck squamous cell carcinoma	497
**Kidney**	898	186	Kidney-ChRCC<br>Kidney-RCC	43<br>143	123	Kidney	123	589	Kidney chromophobe<br>Kidney renal clear cell carcinoma<br>Kidney renal papillary cell carcinoma	51<br>317<br>221
**Liver**	716	313	Liver-HCC	313	57	Liver	57	346	Liver hepatocellular carcinoma	346
**Lung**	1454	78	Lung-AdenoCA<br>Lung-SCC	33<br>45	417	Lung	417	959	Lung adenocarcinoma<br>Lung squamous cell carcinoma	487<br>472
**Lymphoid**	258	196	Lymph-BNHL<br>Lymph-CLL<br>Lymph-NOS	104<br>90<br>2	25	Lymphoid	25	37	Lymphoid neoplasm Diffuse large B-cell lymphoma	37
**Nervous system**	1085	273	CNS-GBM<br>CNS-Medullo<br>CNS-Oligo<br>CNS-PiloAstro	38<br>140<br>18<br>77	71		71	741	Brain Lower Grade glioma<br>Glioblastoma multiforme	434<br>307
**Neuro-endocrine**	281	80	Panc-Endocrine	80	124	Neuro-endocrine	124	77	Adrenocortical carcinoma<br>Pheochromocytoma and paraganglioma	63<br>14
**Ovary**	323	110	Ovary-AdenoCA	110	151	Ovary	151	62	Ovarian serous cystadeno-carcinoma	62
**Pancreas**	465	231	Panc-AdenoCA	231	83	Pancreas	83	151	Pancreatic adenocarcinoma	151
**Prostate**	962	196	Prost-AdenoCA	196	366	Prostate	366	400	Prostate adenocarcinoma	400
**Skin**	688	65	Skin-Melanoma	65	226	Skin	226	397	Skin cutaneous melanoma	397
**Stomach**	518	62	Stomach-AdenoCA	62	41	Stomach	41	415	Stomach adenocarcinoma	415
**Uterus**	477	41	Uterus-AdenoCA	41	57	Uterus	57	379	Uterine corpus endometrial carcinoma	379

Figure S1. Overview of cancer samples and types used in the study. Cancer subtypes (ST) in the three cohorts (TCGA, PCAWG, HMF) were consolidated to 18 cancer types based on anatomical sites.

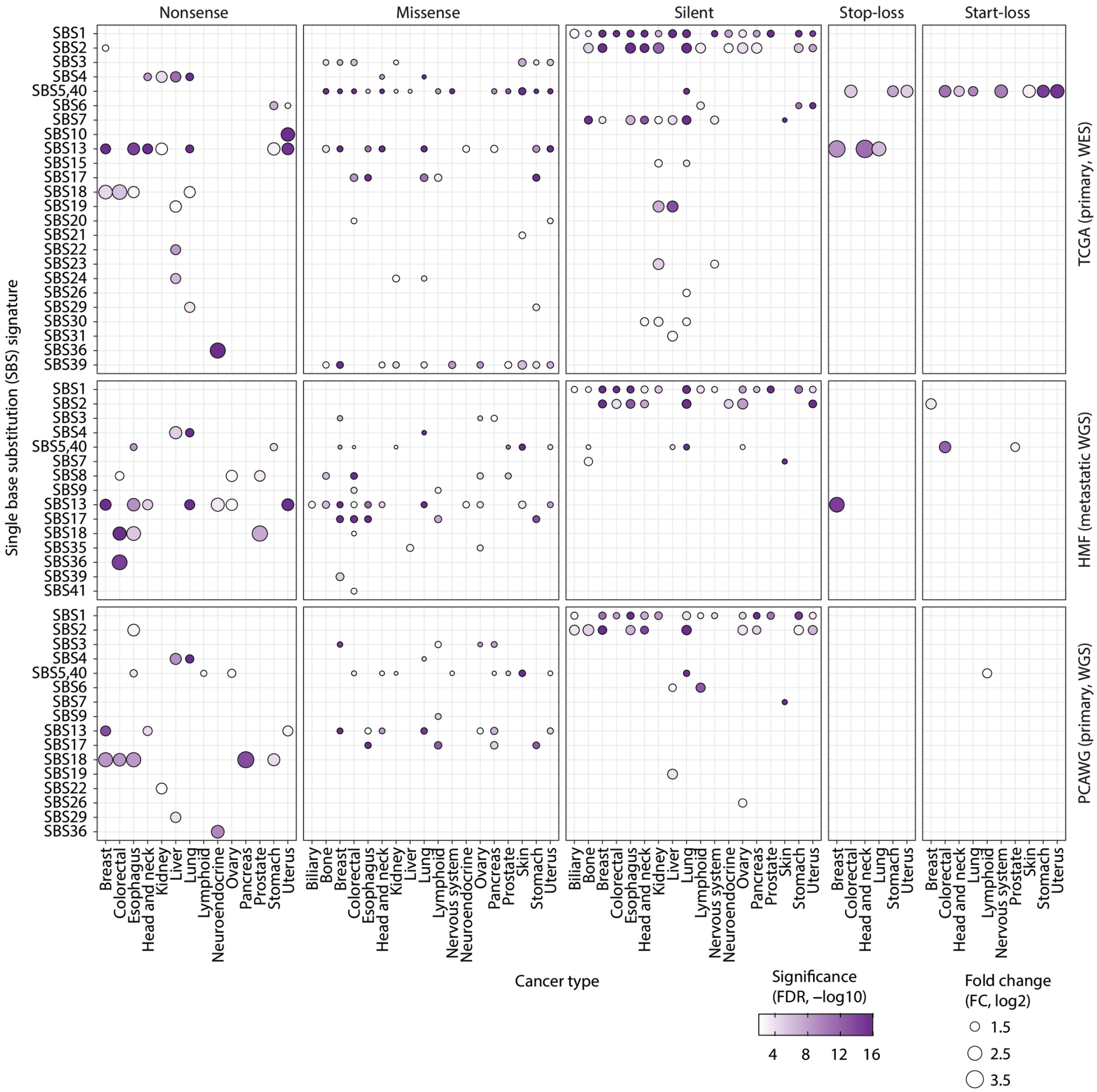

Figure S2. Protein-coding impact of mutational signatures in cancer genomes. Landscape of mutational signatures that are enriched in types of SNVs based on their protein-coding impact. 18 types of primary and metastatic cancers were analysed separately. Significant associations are shown (Fisher's exact test; FDR < 0.01; FDR capped at 10^{-16}). Fold-change (FC) shows the ratio of observed and expected SNV counts.

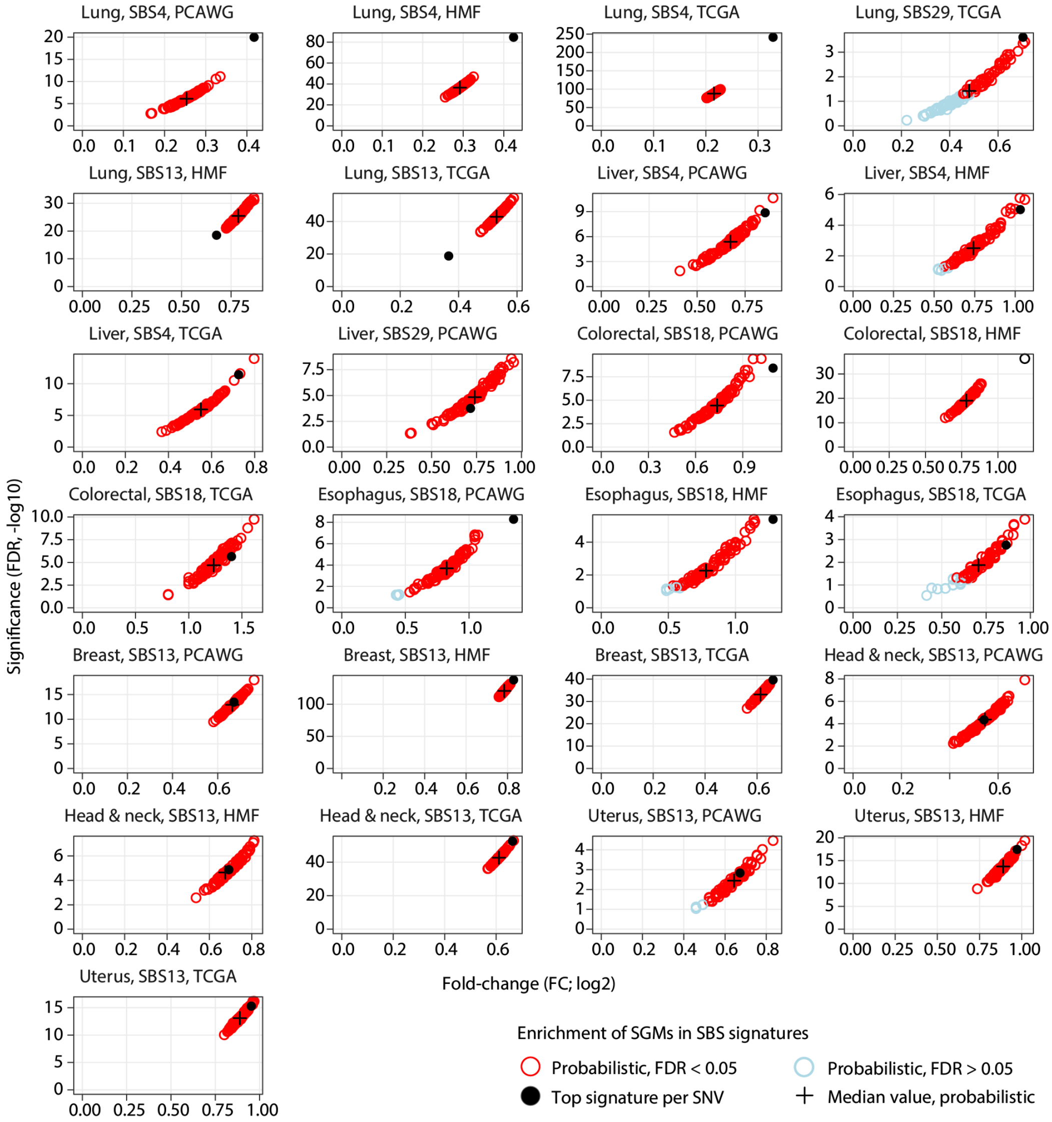

Figure S3. Probabilistic analysis of mutational signature annotations of SNVs and SGM enrichments. Each SNV was assigned randomly to one SBS signature using the multinomial distribution parametrised by all SBS signatures in that cancer sample as well as the trinucleotide context of each SNV, and repeated over 100 iterations (*i.e.*, the probabilistic method). Each resulting set of SNVs was tested for enrichments of SGMs and the selected mutational signatures similarly to the analysis in Figure 1B-C. The probabilistic analysis of SBS signatures confirmed the main analysis of signature annotations of SNVs where each SNV was assigned to the top, most probable SBS signature. The individual enrichments of SGMs in SBS signatures remained highly significant in both approaches.

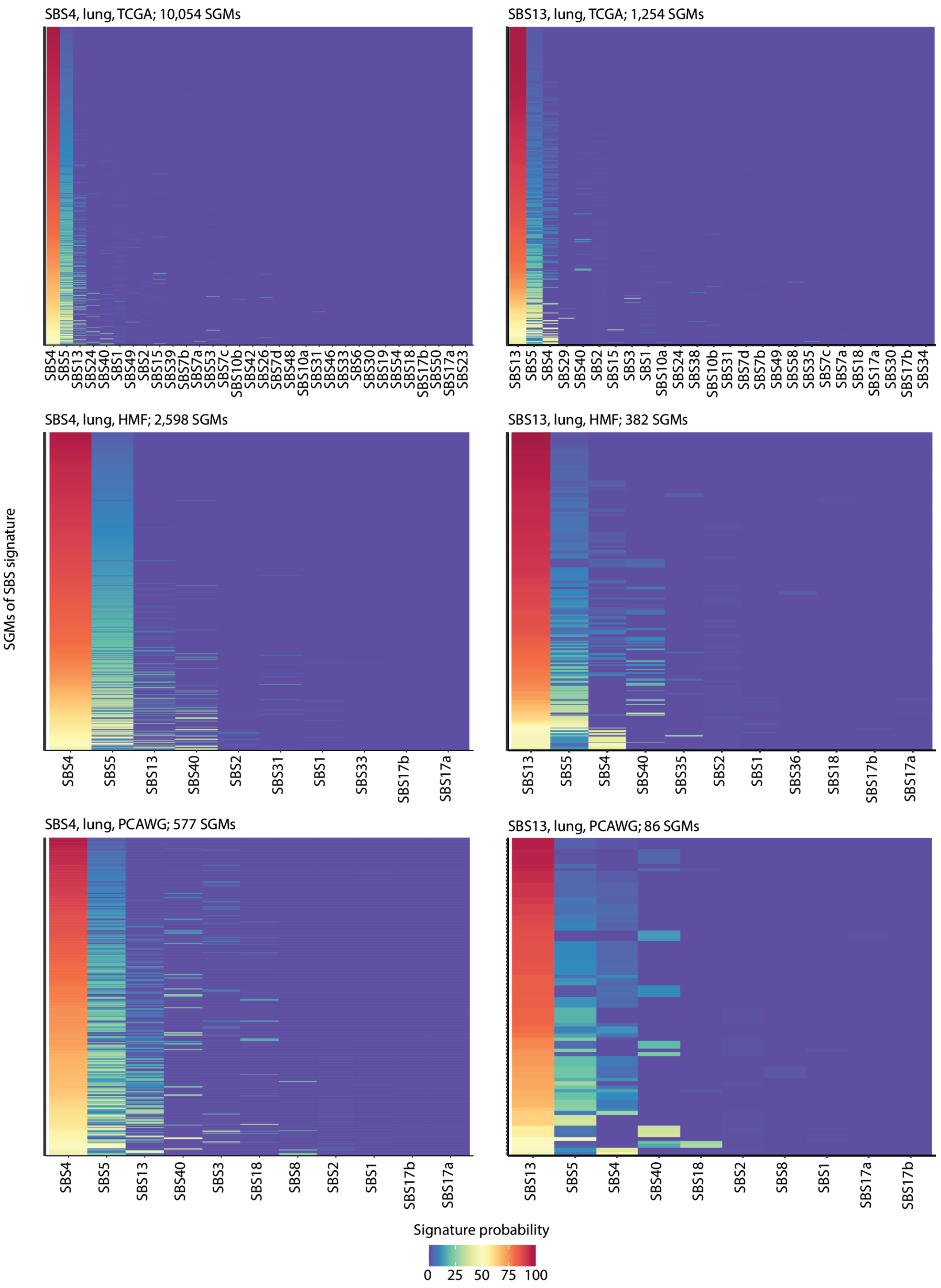

Figure S4. Comparison of signature annotation probabilities of SGMs of the signatures SBS4 and SBS13. In lung cancer, the SNVs are often assigned to either the APOBEC signature SBS13 or the tobacco smoking signature SBS4 such that the two SBS signatures are the less-likely alternatives to each other, together with the clock-like signature SBS5 that has a relatively flat (featureless) profile. The columns representing SBS signatures are ordered by their cumulative probabilities across SGMs.

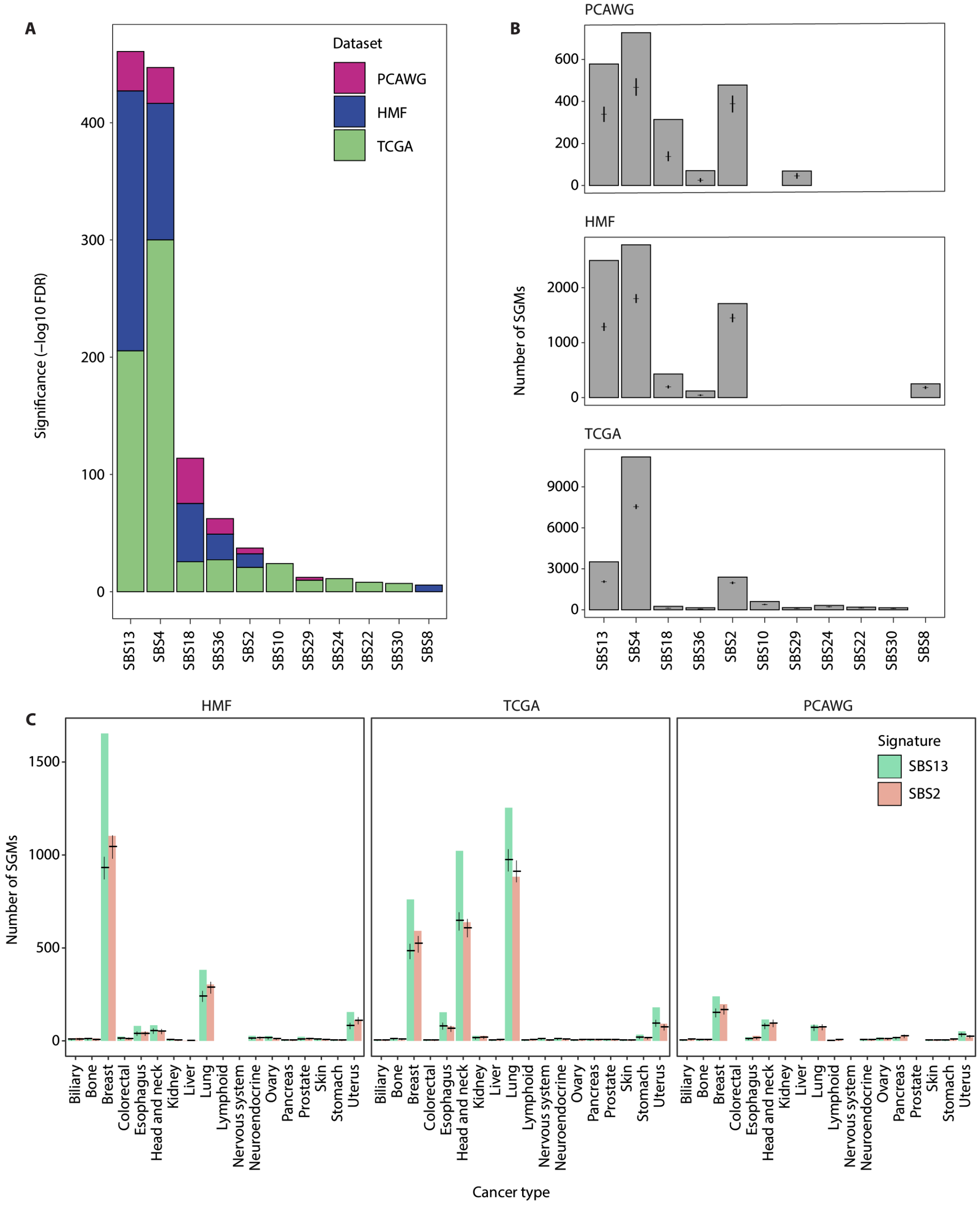

Figure S5. Pan-cancer enrichment of SGMs in mutational signatures. (A) Total significance of SGMs enriched in specific mutational signatures (FDR-adjusted Fisher's exact tests, FDR < 0.01, capped at 10^{-300}). **(B)** Observed SGM of signatures in pan-cancer cohorts. Expected values were derived from binomial distributions. 95% Cis are shown as crosses. **(C)** APOBEC-associated SGMs of the signatures SBS13 and SBS2 in various cancer types. The SBS13 SGMs observed in cancer genomes are substantially more frequent and highly exceed the expected counts of SGMs based on hypergeometric distributions. In contrast, SBS2 SGMs are less frequent overall and the observed SGM counts are close to expected counts. SBS2 and SBS13 co-occur in some cancer types due to high levels of APOBEC activity. The weak pan-cancer enrichment of SBS2 SGMs is likely explained by the increased statistical power to detect small effects in large pan-cancer datasets.

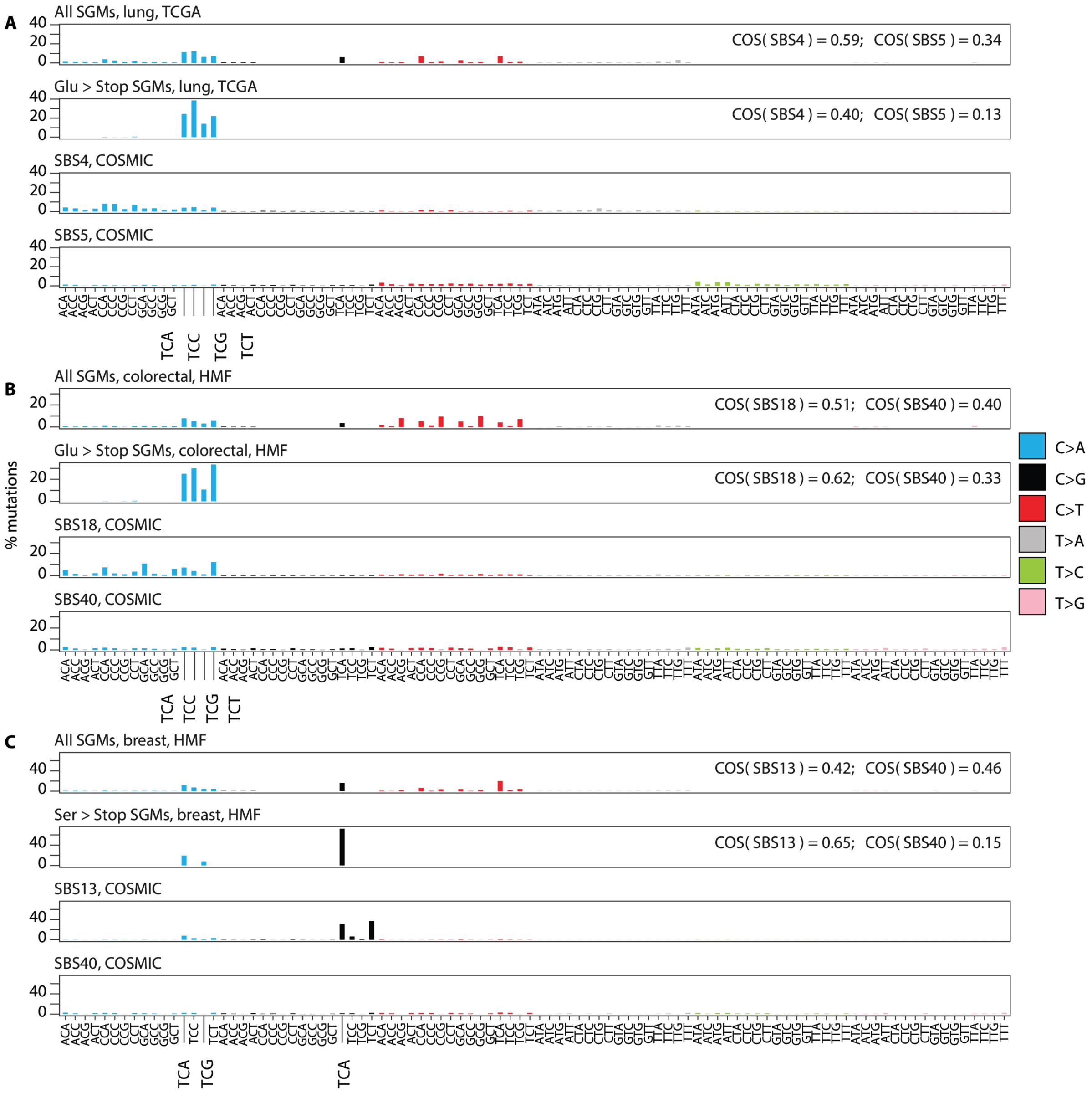

Figure S6. Analysis of COSMIC reference signatures and SGMs. Each panel shows four bar plots of trinucleotide profiles: (i) the observed SGMs, (ii) the SGMs of the most abundant amino acid substitutions (Glu>Stop or Ser>Stop), (iii) the reference COSMIC signatures associated with SGMs (SBS4, SBS18, SBS13), (iv) and the control COSMIC signatures that represent the next most frequent signatures in the respective cancer types (clock-like signatures SBS5 or SBS40). Cosine similarity (COS) scores of SGMs and reference signatures are shown in the top right corners of the bar plots. **(A)** Tobacco signature SBS4 in lung cancer. **(B)** ROS signature SBS18 in colorectal cancer. **(C)** APOBEC signature SBS13 in breast cancer.

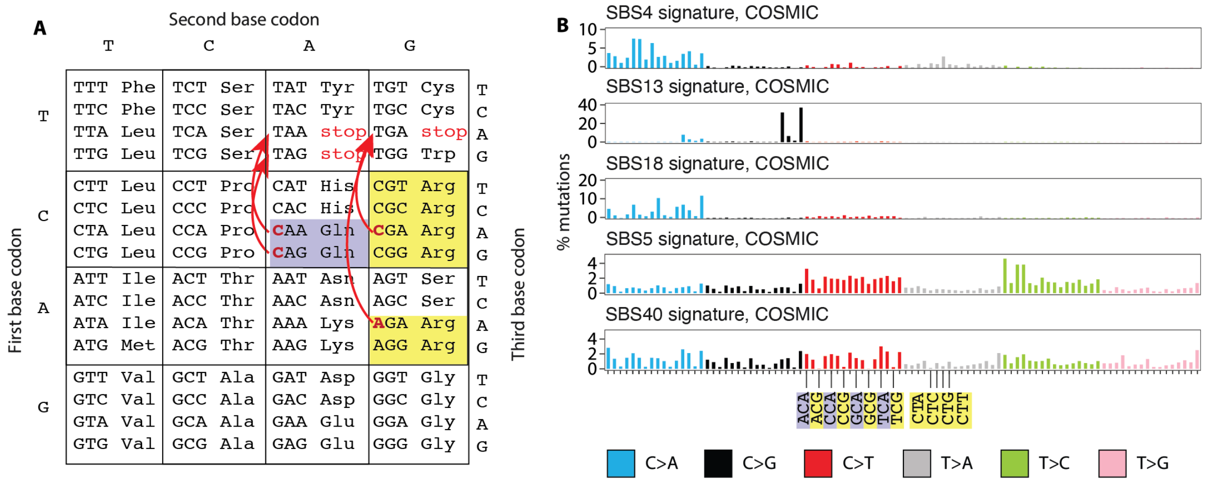

Figure S7. Common SGMs involving arginines and glutamines are incompatible with the signatures SBS4, SBS13, and SBS18. (A) The table highlights the genetic code of common SGMs involving glutamines (Gln>Stop, purple) and arginines (Arg>Stop, yellow). The arrows show all possible SGMs that substitute Arg or Glu codons with stop codons through single base substitutions. **(B)** The reference COSMIC profiles show the trinucleotide preferences of the three mutational signatures enriched in SGMs (SBS4, SBS13, SBS18) and the most common mutational signatures as controls (SBS5 and SBS40). Mutations encoding Gln>Stop and Arg>Stop SGMs are shown. These SGMs often seen in other mutational signatures are not commonly found in the tobacco, APOBEC, and ROS signatures due to their trinucleotide context, and are more likely annotated as the common SBS5 or SBS40 signatures instead.

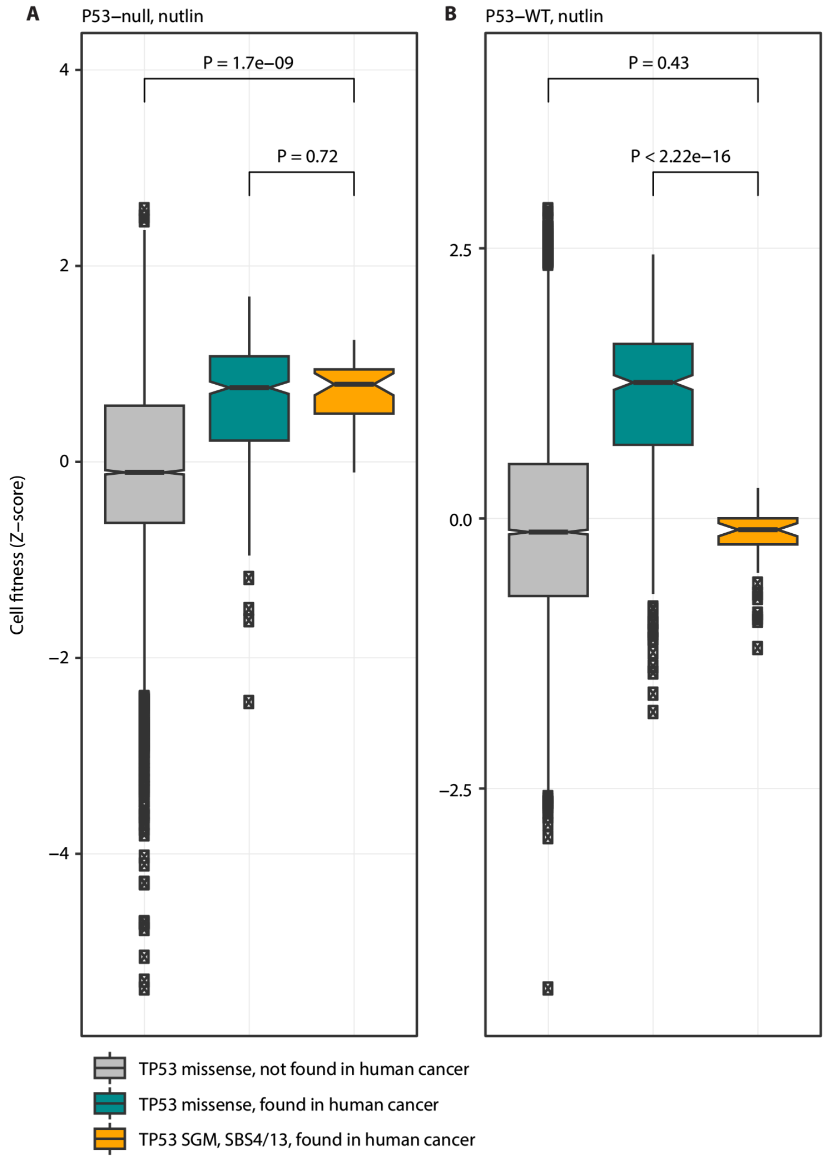

Figure S8. Functional analysis of SGMs in *TP53* using deep mutational scan data. SGMs of SBS4 and SBS13 signatures in *TP53* in human cancer genomes are predicted to have loss-of-function effects, according to deep mutational scanning data from Giacomelli *et al*. (*27*). **(A)** Cell fitness of *TP53*-NULL lung carcinoma epithelial cells (A549) transduced with SBS4/13 *TP53* SGM mutants (yellow), *TP53* missense SNV mutants found in cancer (teal) and all other possible missense SNV mutants (grey) *vs*. A549 cells transduced with wildtype *TP53* upon nutlin treatment, which activates WT *TP53* and results in cell cycle arrest. Cells with SBS4/13 *TP53* SGM mutant and *TP53* missense SNV mutant exhibit higher fitness upon nutlin treatment indicative of p53 loss-of-function. **(B)** In *TP53*-wildtype cells, transduction of cancer-associated *TP53* missense SNVs increases fitness, as most *TP53* mutations found in cancer genomes are dominant negative and therefore inhibit the endogenous WT allele of *TP53*. Transduction of cancer-associated SGMs does not alter the fitness of p53-wildtype A549 cells, as these mutations represent bona fide LOF mutations without dominant negative functions. P-values from Wilcoxon rank-sum tests are shown.

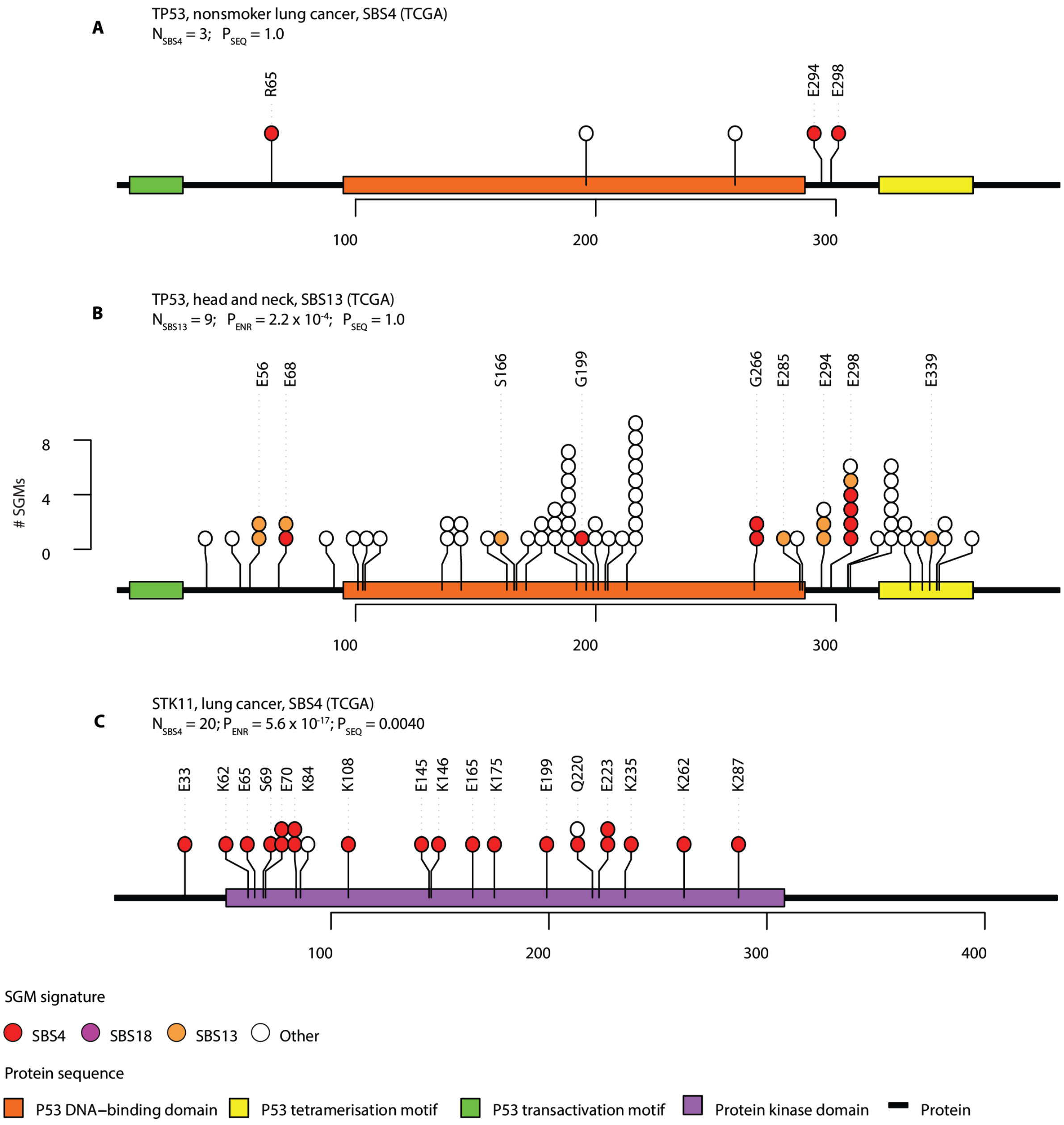

Figure S9. Examples of SGMs in tumor suppressor genes. (A) SGMs in TP53 in 81 lung cancer genomes of patients with no smoking history from TCGA. **(B)** TP53 in 497 head & neck cancers in TCGA includes SGMs of tobacco and APOBEC signatures. **(C)** Most SGMs in STK11 in lung cancer are driven by the tobacco signature SBS4. Circles show all SGMs in the gene and are labelled with the reference residue and the protein sequence position. Colors show the SBS signatures of SGMs (SBS4/13/18; white for others). The title includes the number of SGMs associated with the signature (N_{SBS}), the P-value of signature-associated SGM enrichment (P_{ENR}, Fisher's exact test), and the over-representation of SGMs towards the protein terminus (P_{SEQ}, one-sample Mann-Whitney U-test).

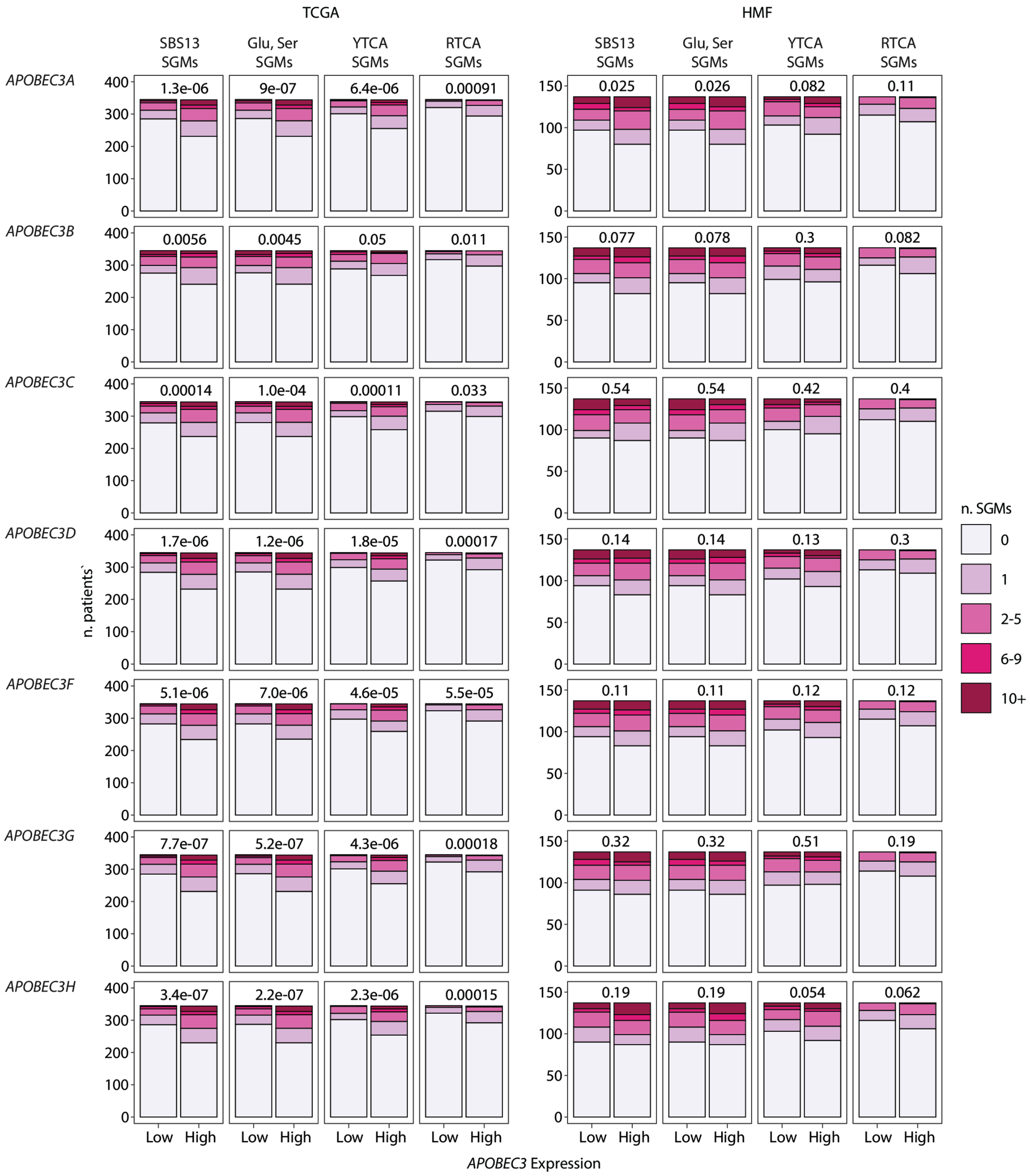

Figure S10. SGM burden in breast cancer associates with increased expression of *APOBEC3* genes. Cancer samples were grouped into two equal groups based on the median expression of each *APOBEC3* gene and different classes of SBS13 SGMs were compared between the groups. Unadjusted Wilcoxon P-values are shown at the top of the bar plots. The attenuated significance apparent in the HMF cohort (right) is potentially due to the smaller cohort sizes and limited matching RNA-seq data in the dataset, resulting in lower statistical power to detect the small effect sizes of relatively infrequent SGMs.

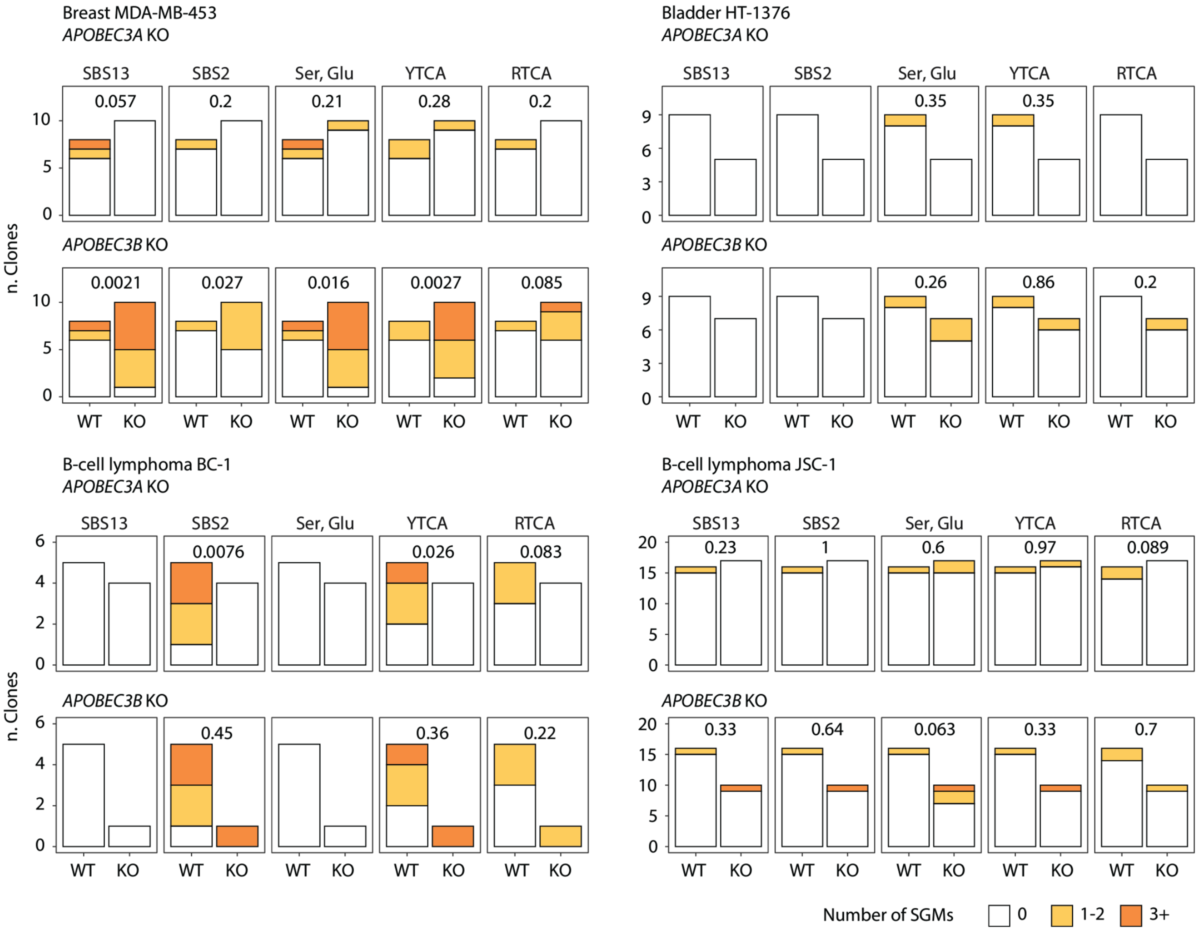

Figure S11. SGM burden in *APOBEC3A/B* knockout (KO) experiments in cell lines. *APOBEC3A* or *APOBEC3B* KO and wildtype cell line clones from the study by Petljak *et al*. (*43*) were compared in the context of SGM burden using negative binomial regression. Unadjusted P-values are shown at the top of the bar plots. In the B-cell lymphoma cell line BC-1, *APOBEC3A* KO cells show a significantly decreased SBS2 SGM burden and fewer SGMs in YTCA motifs. In, the metastatic breast cancer cell line MDA-MB-453, *APOBEC3B* KO cells show a significantly increased SBS13 SGM burden. Another breast cancer cell line BT-474 with a significant SGM decrease in *APOBEC3A* KO cells is shown in Figure 4F. These data provide experimental evidence supporting the hypothesis that SGM burden associates context-specifically with *APOBEC3A* or *APOBEC3B* activity in cancer cell lines.

Table S1A. SGM-enriched genes. List of significant genes from the cancer types and mutational signatures where SGMs occurred more often than expected.

Table S1B. SGM mutations in enriched genes. SGMs in each of the enriched genes.

Table S1C. SGM-enriched pathways. Pathways enriched for the significant genes for the SGMs of SBS4 in lung cancer, SBS4 in liver cancer, SBS13 in breast cancer, SBS13 in head&neck cancer, and SBS13 in uterine cancer. For the cancer type contributions, a value of 1 indicates that the pathway was enriched in that cancer type individually, and a value of 0 indicates that pathway was not enriched in that cancer type individually. A value of 1 in the combined column indicates that the pathway was not enriched in any cancer type individually and was only enriched when all cancer types were jointly considered.
